# Structure and Nitrite Reductase Activity of the Di-iron Protein ScdA in *Staphylococcus aureus*

**DOI:** 10.1021/jacs.5c05573

**Published:** 2025-08-22

**Authors:** Hung-Ying Chen, Ruei-Fong Tsai, Yi-Shan Lu, Yang-Chun Cheng, Hsiang-Yuan Fan-Chiang, Chu-Ya Wu, Feng-Chun Lo, Hsuan-Wei Kuo, Wei-Kai Yang, Wan-Yi Liao, Nien-Jen Hu, Shih-Che Sue, Yun-Wei Chiang

**Affiliations:** † Department of Chemistry, 34881 National Tsing Hua University, Hsinchu 300-044, Taiwan; ‡ Institute of Bioinformatics and Structural Biology, National Tsing Hua University, Hsinchu 300-044, Taiwan; § Graduate Institute of Biochemistry, 34916 National Chung Hsing University, Taichung 402-202, Taiwan

## Abstract

Pathogenic *Staphylococcus aureus* endures bursts of host-derived reactive nitrogen species, yet the molecular defenses that enable this resilience have remained unclear. We now show that the previously enigmatic di-iron enzyme ScdA functions as a nitrite reductase, converting nitrite to nitric oxide (NO), and we elucidate the structural elements that support this activity. Using an integrative toolkit—X-ray crystallography, solution NMR, AlphaFold modeling, and pulsed EPR/DEER—we solved the full-length homodimeric structure of ScdA and identified a robust di-iron center that forms stable iron-nitrosyl intermediates. Targeted mutagenesis reveals that redox-active cysteines and dimerization state tune catalytic output, whereas steady-state kinetics confirm efficient nitrite-to-NO turnover. In vivo, ScdA overexpression in *Escherichia coli* suppresses growth under nitrite-rich conditions, highlighting the cytotoxic potency of the NO it generates. By coupling structure to function, our work clarifies *S. aureus* strategies for managing nitrosylative stress and points to ScdA as a potential vulnerability in antibiotic-resistant pathogens.

## Graphical abstract

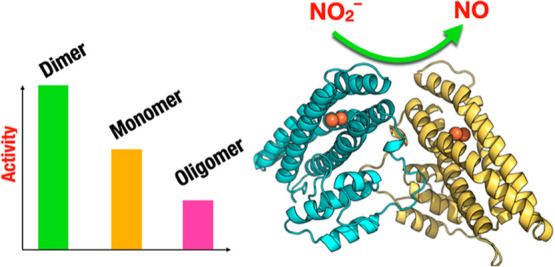

## Introduction

*Staphylococcus aureus* is a leading human pathogen implicated in a broad range of infections, from benign skin lesions to severe diseases such as pneumonia, endocarditis, and sepsis. The escalating prevalence of antibiotic-resistant strains, particularly methicillin-resistant *S. aureus* (MRSA), highlights the critical need for alternative therapeutic options.^1,2^ A hallmark of *S. aureus* pathogenicity is its remarkable capacity to withstand host-imposed stresses. Among the most potent of these are reactive oxygen species (ROS) and reactive nitrogen species (RNS), which are deployed by immune cells to damage bacterial proteins, lipids, and nucleic acids.^3,4^ The resilience of *S. aureus* in the face of such challenges underscores its evolution of multifaceted stress-response mechanisms.

To counter these threats, *S. aureus* encodes an array of antioxidant and antinitrosylative defenses, including stress-responsive proteins such as ScdA. When first described, ScdA was placed in the so-called “repair of iron centres” (RIC) family and proposed to protect iron–sulfur enzymes that are damaged by ROS or RNS.^5^ Its expression is regulated by the two-component system SrrAB, which becomes activated under oxidative or nitrosylative stress conditions.^6^ Notably, ScdA has been shown to restore the activities of iron-sulfur cluster enzymes (e.g., aconitase and fumarase) following their inactivation by reactive species.^5^ Despite these advances, many details regarding ScdA’s broader roles remain largely unknown, and the structural underpinnings of its protective functions have yet to be fully delineated.

Within the RIC family,^7,8^ homologues such as YtfE in *Escherichia coli* have gathered attention for their ability to repair iron-sulfur clusters and mitigate nitrosylative stress.^9,10^ YtfE has been shown to reduce both nitric oxide (NO) and nitrite, indicating a capacity to detoxify reactive species or generate reactive intermediates under specific conditions.^11–16^ A 2022 study, supported by in vivo data, established that YtfE is a highly active nitrite reductase that promotes NO formation, confirming nitrite reduction as its primary physiological role.^11^ Whether ScdA in *S. aureus* undertakes analogous functions has not been conclusively determined, leaving a critical gap in our understanding of ScdA’s potential enzymatic repertoire. Moreover, while some studies suggest that ScdA helps the bacterium withstand RNS, the possibility that it may also generate reactive intermediates, most notably NO, naturally prompts the question of how *S. aureus* manages or benefits from what is typically a cytotoxic molecule. The presence of one or more regulatory mechanisms (e.g., compartmentalization, scavenging enzymes, or tightly controlled expression) could allow *S. aureus* to harness small or localized pools of NO in ways that remain to be fully elucidated, adding further intrigue to ScdA’s role in bacterial stress responses.

In this study, we address these questions by presenting the first full-length structure of ScdA. Using a comprehensive approach combining AlphaFold-3 (AF) modeling,^17^ X-ray crystallography, NMR spectroscopy, and double electron–electron resonance (DEER),^18^ we delineate the structural and conformational features that define ScdA’s function. Our findings demonstrate that ScdA harbors a di-iron catalytic center, consistent with its placement in the RIC family. Moreover, we show that ScdA catalyzes the reduction of nitrite to NO, as verified by chemical assays and electron paramagnetic resonance (EPR) spectroscopy. Although NO is typically cytotoxic, *S. aureus* may exploit additional pathways or mechanisms that either neutralize excess NO or harness it for specialized processes, possibilities that remain to be explored in detail. From a therapeutic standpoint, the discovery that ScdA can generate a potent antimicrobial species highlights its potential as a target for new interventions against MRSA and other resistant variants. By elucidating how this essential stress-response protein balances protective and potentially deleterious functions, our work provides an important foundation for future efforts to manipulate ScdA toward clinical benefit.

## Results and Discussion

We first demonstrated the successful overexpression and purification of *S. aureus* ScdA. SDS–PAGE analysis of the purified wild-type (WT) ScdA revealed an apparent molecular weight of approximately 27 kDa (Figure 1A), consistent with the expected value. Subsequent size-exclusion chromatography (SEC) indicated that purified WT ScdA (as-isolated) eluted in two distinct peaks at 9.2 and 10.9 mL (Figure 1B), suggesting oligomeric and dimeric states. The native ScdA sequence has three cysteine residues (Cys30, Cys31, and Cys191), which may form intermolecular disulfide bonds and thereby promote oligomerization.

**Figure 1:**
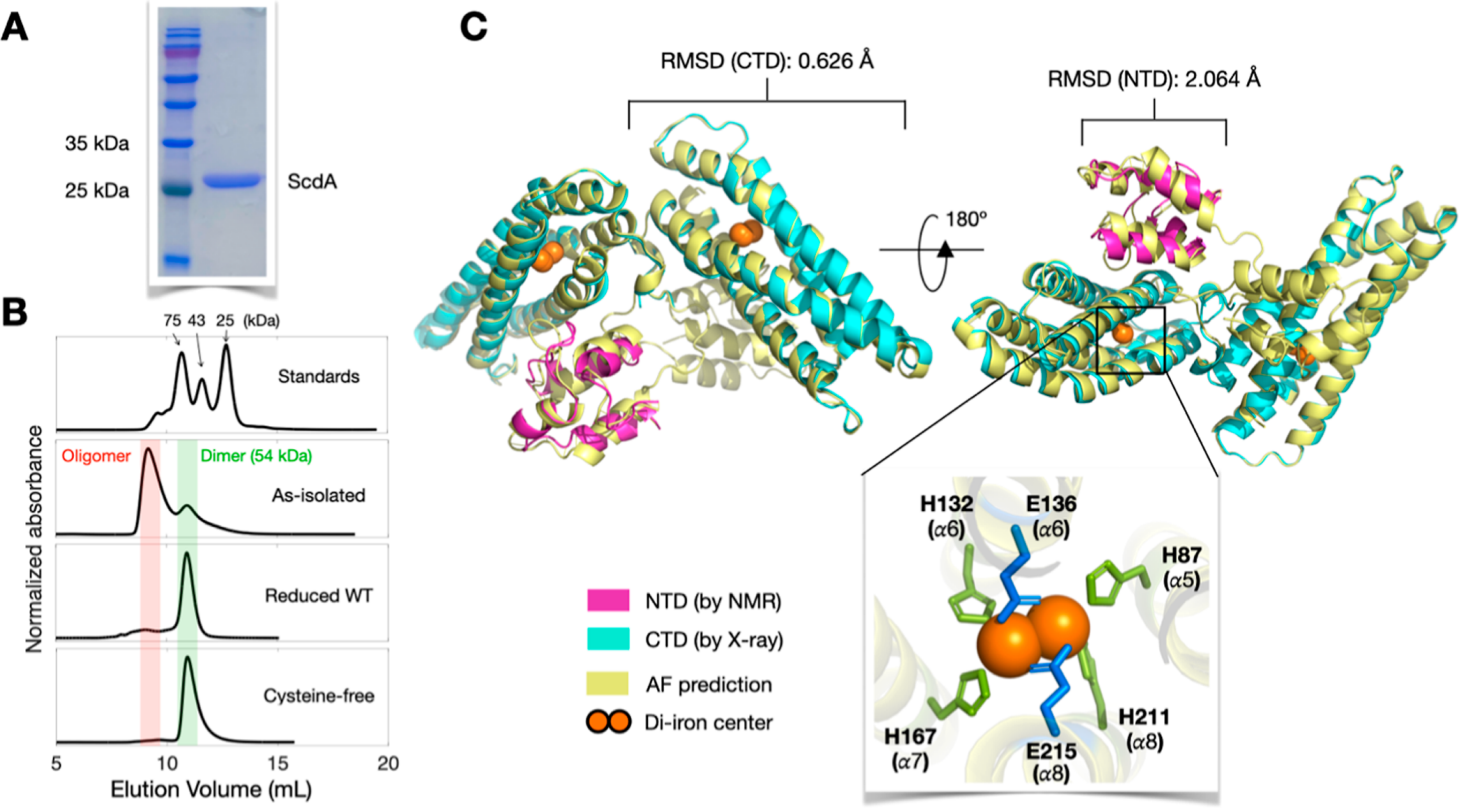
Purification and Structural Determination of ScdA. (A) SDS–PAGE analysis confirming the purity of recombinant ScdA; the protein carries an N-terminal 6 × His tag. (B) SEC profiles showing that as-isolated ScdA exists as a mixture of oligomers and dimers, whereas DTT-reduced ScdA and the CF variant predominantly form dimers (∼54 kDa). (C) Superposition of structural models derived from NMR (NTD), X-ray crystallography (CTD), and AlphaFold predictions, demonstrating good alignment (low RMSD) for both domains. The six di-iron coordinating residues are H87, E132, E136, H167, E211, and E215. See also Figures S1–S3 for additional details on the X-ray (PDB: 9J47), sequence alignment, and NMR data.

To investigate the role of these cysteines, we treated WT ScdA with the reducing agent dithiothreitol (DTT). Under reducing conditions, the SEC fraction corresponding to the oligomeric state (elution at 9.2 mL) declined significantly, whereas the fraction representing the dimeric state (10.9 mL) increased. We also analyzed a cysteine-free (CF) variant (C30A/C31A/C191A) by SEC. This CF variant produced a single peak at 10.9 mL, confirming that ScdA is a homodimer in the absence of intermolecular disulfide bonds.

### Determination of the Dimeric ScdA Structural Model Using a Combined Approach

To elucidate the structure of dimeric ScdA, we employed a strategy integrating X-ray crystallography, NMR spectroscopy, and AlphaFold-3 (AF) prediction (Figure 1C). The high-resolution X-ray crystal structure of CF ScdA revealed a C-terminal domain (CTD) adopting a four-α-helix bundle (Figures 1C and S1). However, the N-terminal domain (NTD) could not be resolved, presumably due to low electron density in that region, suggesting that the NTD may be relatively flexible or disordered. The crystal structure demonstrated that the CTD forms a homodimer, with each monomer encompassing a hemerythrin-like domain that binds nonheme iron. The dimer interface is located between two intermolecular α-helices (helix 5). We will discuss the interface interactions in a subsequent section. Based on the crystal structure, residues H87, H132, E136, H167, H211, and E215 were identified to coordinate the di-iron center (Figure 1C). These residues are highly conserved among ScdA homologues (Figure S2).

Because X-ray crystallography did not resolve the NTD, we purified an NTD-only variant of CF ScdA (residues 1–63) and determined its structure by NMR spectroscopy (Figure S3). This fragment adopts a globular fold composed of four short α-helices. To generate the full-length model, we employed AF to predict the complete protein conformation (Figure 1C). Notably, the AF model also proposes a dimer assembly with a CTD arrangement closely mirroring that observed in the crystal structure. We then superimposed our crystal-derived CTD and NMR-derived NTD structures onto the AF model, yielding root-mean-square deviations (RMSDs) of 2.06 Å for the NTD and 0.626 Å for the CTD, evidence of substantial structural agreement. Consequently, we regard the AF prediction as a robust representation of the dimeric ScdA.

Although X-ray crystallography and NMR provide high-resolution structural snapshots, they give limited direct information on intersubunit distances in solution; therefore, we employed DEER spectroscopy with site-directed spin labeling to examine the dimeric arrangement of ScdA.^19–21^ We engineered 12 single-labeled CF variants, with three labeled sites in the NTD and nine in the CTD (Figure 2A), ensuring that each dimer contained exactly two spin labels. By analyzing the DEER time-domain data (Figure S4), we generated interspin distance distributions (Figure 2B) and compared them with distributions simulated from the AF model using the chiLife program.^18,22–24^ These comparisons showed good overall agreement, indicating that the AF-based structure closely approximates the ScdA dimer in solution. For the 42- and 95-labeled variants we observed almost featureless DEER traces whose first detectable dipolar modulation would occur beyond our 3 μs acquisition window, corresponding to interspin separations longer than ∼6 nm. For the remaining 10 variants, the most probable distances corresponded closely (<0.3 nm) with AF-based estimates, and the crystal and NMR structures showed similar correspondence. Notably, the DEER-derived distributions were generally broader than those from AF simulations, implying greater conformational heterogeneity in solution, particularly in the NTD (e.g., at sites 11 and 53 in Figure 2B). These observations corroborate the conformational heterogeneity in the NTD suggested by X-ray crystallography for the NTD. Collectively, these results confirm that the AF model provides a credible representation of homodimeric ScdA while also highlighting its dynamic nature, especially in the N-terminal region.

**Figure 2:**
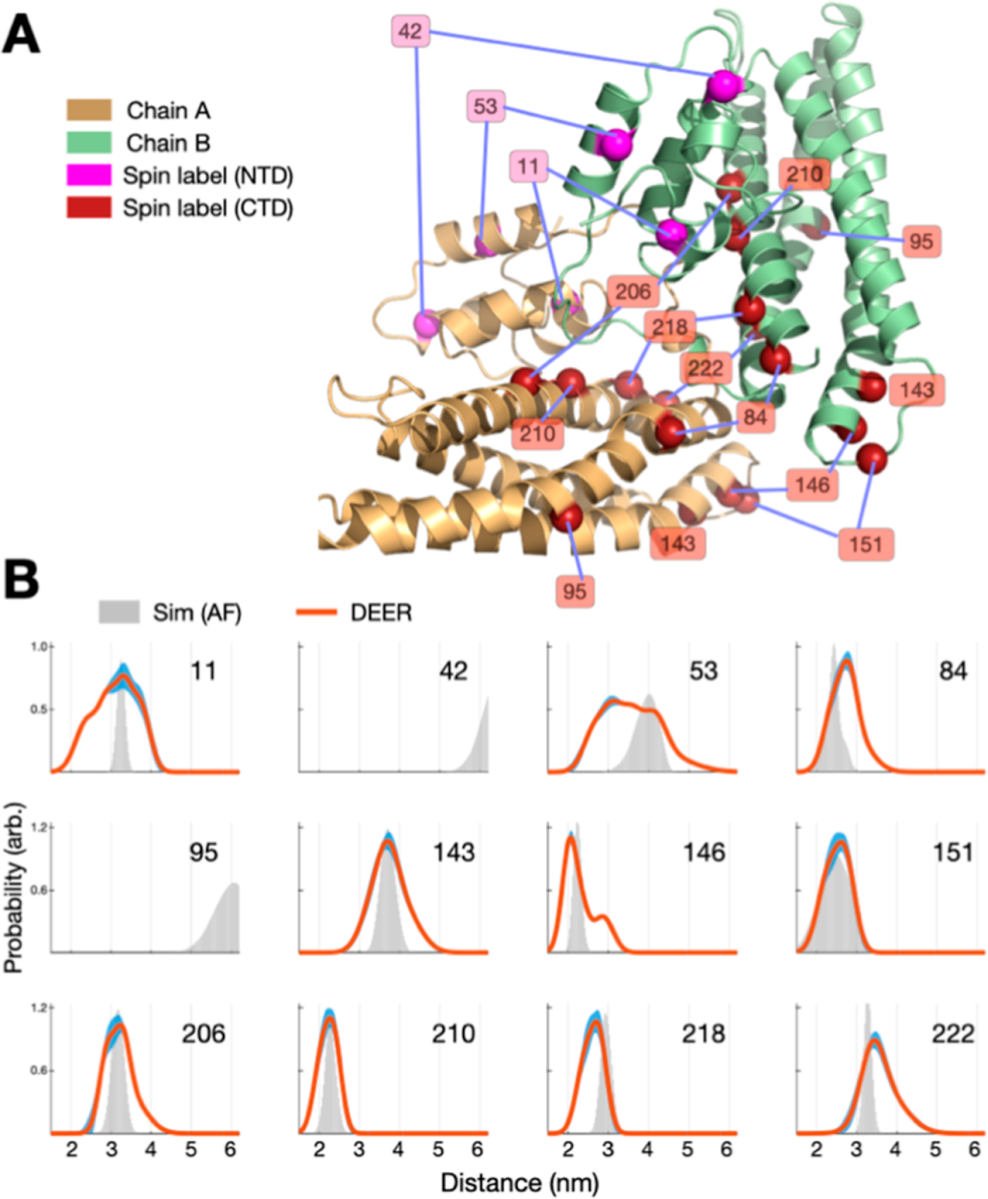
Probing Dimer Conformational Dynamics Using DEER. (A) Schematic illustrating the spin-labeling sites selected for DEER measurements on ScdA dimers. (B) DEER-derived distance distributions for singly labeled ScdA variants, with shading (blue) representing ±2 standard deviations. Simulations (gray) based on the AF model generally concur with experimental data. Variants labeled at positions 42 and 95 display interspin distances exceeding the instrument detection limit. Overall, the results support the AF-based conformation of the ScdA dimer. See also Figure S4 for raw DEER data.

### Identification of the Dimer Interface

To explore the details of ScdA dimerization, we focused on the interface between the two monomers. From the X-ray result, we observed that residues near Ser77 (S77), particularly S77 and Gln80 (Q80), appear to form a hydrogen-bonding network that brings the two monomers into close proximity (Figure 3A). To verify these interactions, we prepared various CF ScdA mutations and assessed their oligomeric states via SEC.

**Figure 3:**
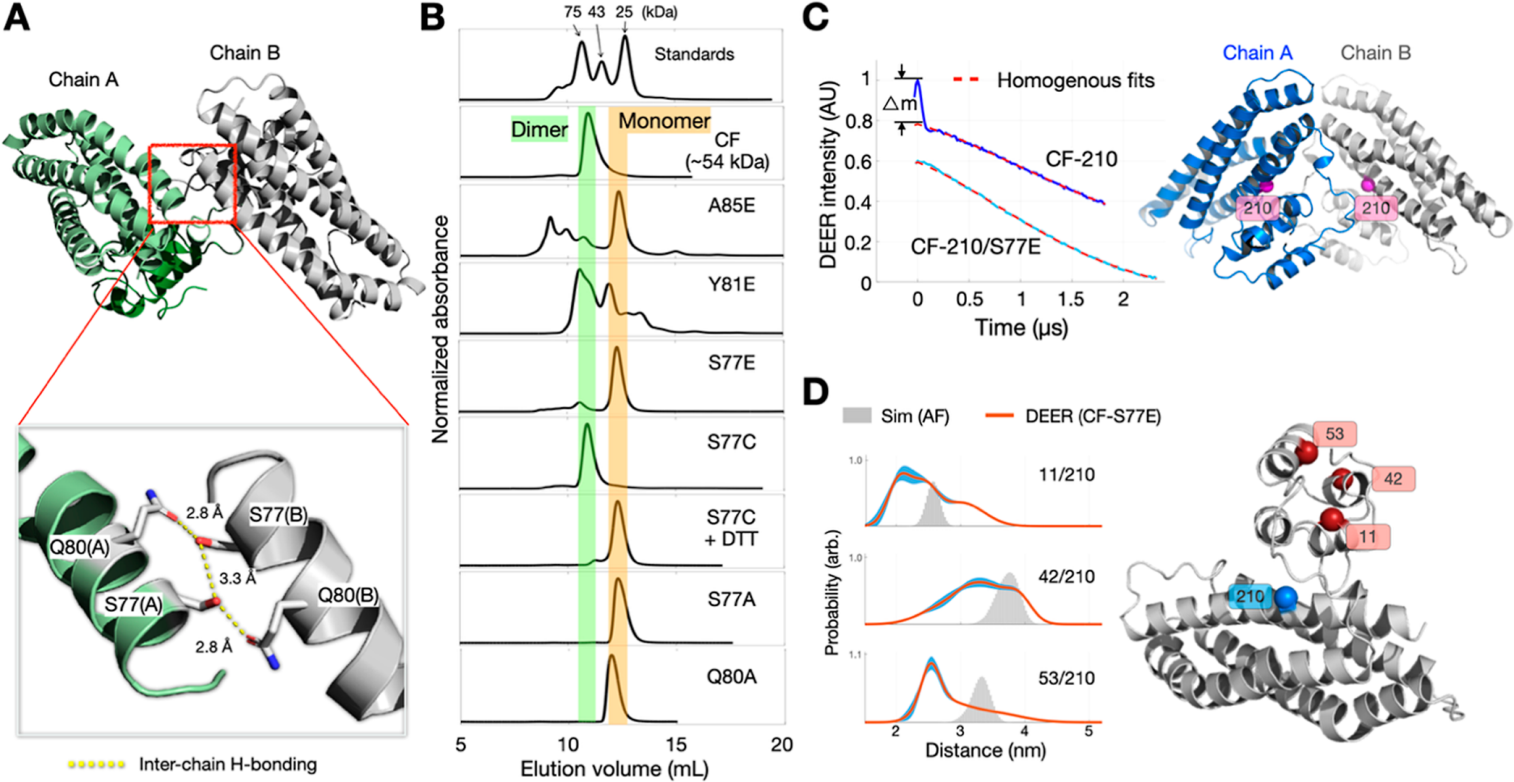
Investigating the ScdA Dimer Interface. (A) Structural highlight of key residues (S77 and Q80) proposed to retain dimer formation through intermonomer hydrogen bonding. The hydrogen bonding network can be observed in both the crystal and AF results. (B) SEC profiles of CF ScdA mutants carrying substitutions near the interface. CF was used to prepare the mutations shown. Only mutations at S77 and Q80 effectively disrupt dimerization, yielding monomeric protein. (C) DEER measurements for CF variants spin-labeled at site 210. The CF-210 mutant shows distinct dipolar modulation (Δm), evidence for the dimer formation. The CF-210/S77E mutant displays negligible dipolar modulation, indicating that S77E disrupts dimer formation and stabilizes a monomeric state. (D) DEER data for doubly labeled CF-S77E variants (predominantly monomeric), with spin labels positioned in the NTD and CTD, respectively. Spin-labeling positions are indicated in the plot. Distance distributions are significantly broader than the AF-based simulations (gray), suggesting monomeric ScdA is in a much wider ensemble of conformations.

We first substituted individual residues with glutamic acid (E) to exploit electrostatic repulsion that could disrupt the interface. Variants S77E, Y81E, and A85E showed substantial changes in elution profiles. Notably, S77E yielded a purely monomeric peak, whereas Y81E and A85E gave mixtures of oligomers, dimers, and monomers. These contrasting outcomes highlight the central importance of S77 in maintaining the dimeric state: Ser77 residues from each monomer directly face one another at the core of the interface, forming intermonomer contacts essential for stable dimer formation. When replaced by a negatively charged glutamate at site S77, these interactions are particularly disrupted, driving the entire protein population into monomeric form. By contrast, Y81 and A85, though located near the interface, are less crucial to its central architecture, so their glutamate substitutions partially destabilize the interface but do not completely abolish dimerization.

We further investigated this residue by preparing S77C and S77A variants. The S77C mutant primarily formed dimers, but they dissociated into monomers in the presence of DTT, implying that an intermolecular disulfide bond formed at position 77. In contrast, S77A produced only monomers in SEC elution (Figure 3B), suggesting that removal of the hydroxyl group disrupts hydrogen bonding at the interface. Similarly, the Q80A mutation favored monomer formation, indicating that Q80 also contributes to dimer stabilization. We conclude that an intermolecular hydrogen-bonding network formed by S77 and Q80 residues on each monomer underpins ScdA dimer assembly.

Although serine–serine hydrogen bonding between helices is relatively rare, it can be functionally significant in certain contexts.^25,26^ To verify the critical role of S77 in dimerization, we also carried out additional DEER spectroscopy. A CF variant spin-labeled at site 210 (CF-210) demonstrated characteristic dipolar oscillations associated with dimeric proteins (Figure 3C). However, the analogous construct harboring S77E (CF-210/S77E) displayed a monotonically decaying DEER trace lacking dipolar modulation, consistent with a fully monomeric distribution. These data reinforce that the hydrogen-bonding network featuring S77 and Q80 is essential for dimer formation.

With CF-S77E effectively locking ScdA in a monomeric state, we next examined the relative positions of the NTD and CTD in a single ScdA subunit by engineering doubly spin-labeled variants 11/210, 42/210, and 53/210 (Figure 3D). DEER-derived distance distributions for these CF-S77E mutants were significantly broader than those predicted by the AF model, indicative of a much wider ensemble of ScdA conformations in the absence of dimerization. Nevertheless, the broad distributions still encompassed the simulated distances. Overall, these results confirm that when ScdA is monomeric, it retains a general structural framework similar to AF predictions but exhibits higher conformational dynamics.

### Investigation of ScdA’s Novel Function as a Nitrite Reductase

Beyond delineating ScdA’s structure, we discovered that ScdA serves as a nitrite reductase, converting nitrite to NO. We performed UV–vis spectroscopy on three sample sets: as-isolated WT, DTT-reduced WT (referred to as reduced WT), and reduced WT treated with nitrite (Figure 4A). The as-isolated WT displayed broad absorptions at 350 and 522 nm, mirroring the mixed-valence Fe­(II)–Fe­(III) centers reported for *E. coli* YtfE, a homologous di-iron protein.^11,12^ Reduction by DTT decreased these peak intensities, indicating conversion to a diferrous Fe­(II)–Fe­(II) state. When nitrite was added to the reduced WT, a prominent peak emerged at 397 nm. This feature corresponds to the formation of an iron-nitrosyl complex, demonstrating that ScdA reduces nitrite to NO, which then binds to the di-iron center.^11,14,27^ Additional UV–vis measurements on ScdA variants are provided in the Supporting Information (Figure S5A).

**Figure 4:**
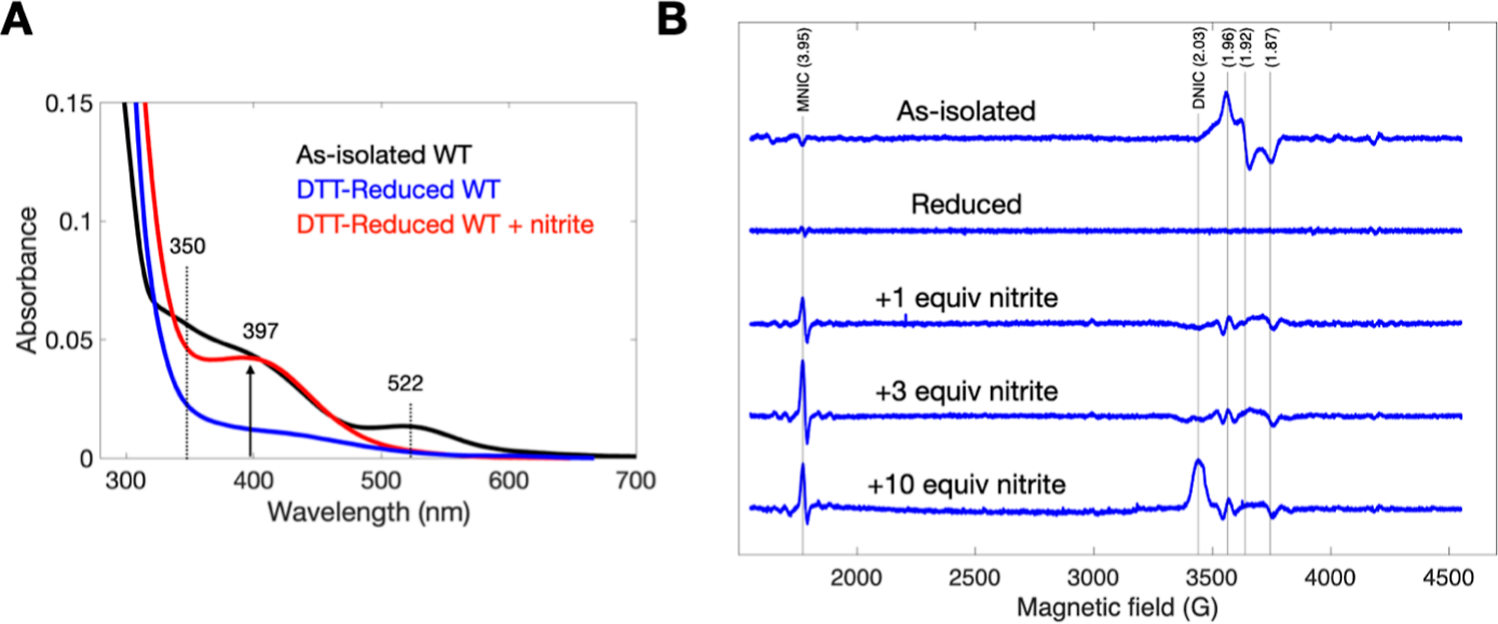
UV–Vis and EPR Characterization of ScdA. (A) UV–Vis spectra of as-isolated (mixed-valent) WT ScdA (black), DTT-reduced WT ScdA (blue), and DTT-reduced WT ScdA treated with excess nitrite (red). The appearance of a 397 nm band upon nitrite addition indicates iron-nitrosyl complex formation in the reduced protein. (B) EPR spectra at 10 K for as-isolated WT ScdA, as well as DTT-reduced WT ScdA treated with 1, 3, or 10 equivalents of nitrite. Signals corresponding to mixed-valent ScdA (*g* = 1.96, 1.92, 1.87), the mononitrosyl iron complex (MNIC, *g* = 3.95), and the dinitrosyl iron complex (DNIC, *g* = 2.03) are noted. See also Figure S5 for UV–Vis spectra of additional variants.

EPR spectroscopy at 10 K further confirmed this activity (Figure 4B). The as-isolated WT displayed resonances at *g* = 1.96, 1.92, and 1.87, characteristic of a mixed-valence Fe­(II)–Fe­(III) di-iron center.^5,11,12^ Treatment with DTT rendered the sample EPR silent, consistent with a diamagnetic Fe­(II)–Fe­(II) state. Introducing one equivalent of nitrite to the reduced ScdA generated a major peak at *g* = 3.95 and a minor peak near *g* = 2, hallmarks of a mononitrosyl iron complex (MNIC). Increasing nitrite to three equivalents enhanced the MNIC peaks. However, adding ten equivalents shifted the spectrum to include a strong dinitrosyl iron complex (DNIC) signal at *g* = 2.03, signifying the accumulation of NO and subsequent formation of DNIC in the presence of excess reducing agent DTT. These findings confirm that ScdA functions as a nitrite reductase, producing NO at its di-iron center.

### Kinetic Study of ScdA’s Nitrite Reduction Activity

To characterize the nitrite reductase kinetics of ScdA, we adapted a previously described methyl viologen (MV) consumption assay used for YtfE-mediated nitrite reduction.^11^ In this system, the oxidation of MV cation radicals (MV^•+^) proceeds at a 1:1 stoichiometric ratio with the reduction of nitrite, allowing MV to serve as a convenient reporter for catalytic turnover. Under anaerobic conditions, we incubated ScdA (1 μM) with MV cation radicals in the presence of increasing concentrations of nitrite (1–1000 μM) and monitored the decrease in absorbance at 600 nm (OD_600_), which reflects MV oxidation over time (Figures 5 and S6).

**Figure 5:**
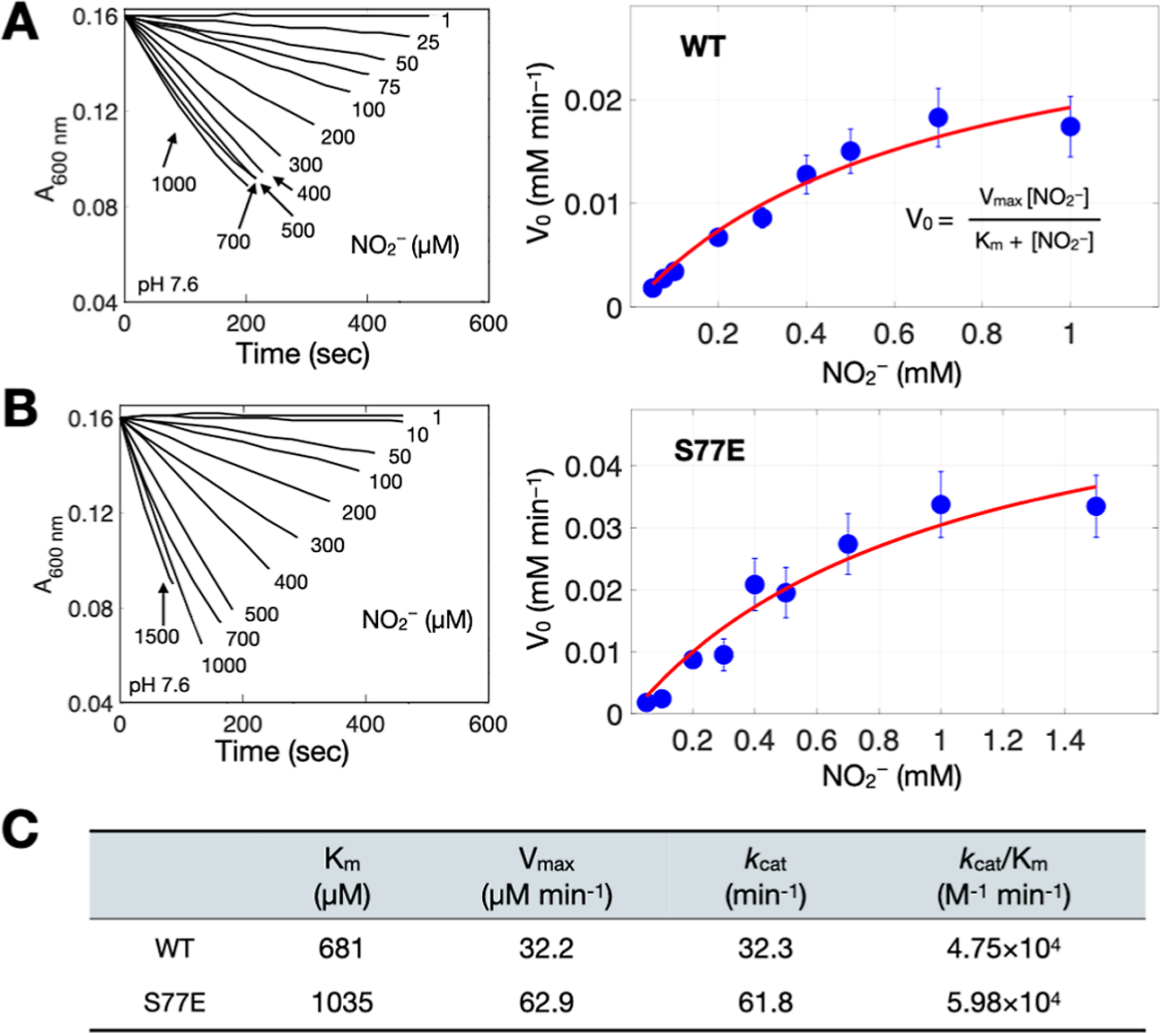
Kinetics of ScdA-Mediated Nitrite Reduction. (A) Left: Time-dependent oxidation of MV cation radicals by ScdA (1 μM) under varying nitrite concentrations (1–1000 μM) for WT ScdA at pH 7.6, monitored at 600 nm. Right: Initial rates (*V*_0_) fitted with the Michaelis–Menten equation (red line). (B) Methyl viologen oxidation (left) and V_0_ analysis (right) for the S77E variant. Error bars represent ±standard deviation. (C) Kinetic parameters for WT and S77E, including Michaelis constants and turnover rates. The estimated uncertainty for the reported kinetic parameters is ±5%, based on error propagation from multiple independent measurements (*n* ≥ 3). See also Figures S6 and S7 for additional methyl viologen assay data and measurments of iron content, respectively.

By plotting the initial rates (*V*_0_) as a function of nitrite concentration and fitting these data to the Michaelis–Menten model, we derived kinetic parameters for WT ScdA (Figure 5A): a Michaelis constant (*K*_m_) of 681 μM, a turnover number (*k*_cat_) of 32.3 min^–1^, and a catalytic efficiency (*k*_cat_/*K*_m_) of 4.75 × 10^4^ M^–1^ min^–1^. Control experiments verified that MV cation radicals remained stable under anaerobic conditions and was not consumed by nitrite in the absence of ScdA (Figure S6). We then assessed the S77E mutant, which disrupts the dimeric state, and found that although it retains nitrite reductase activity, its kinetic profile differs from WT (Figure 5B,C). Specifically, S77E exhibits a ∼1.5-fold higher *K*_m_ (indicating weaker substrate affinity) yet approximately doubles the turnover rate (*k*_cat_). Consequently, its overall catalytic efficiency (*k*_cat_/*K*_m_) increases by ∼25% relative to WT, suggesting that diminished substrate binding is counterbalanced by an enhanced catalytic turnover. Additionally, the MV-based assay showed that mutating any one of the six di-iron-coordinating residues abolishes nitrite reduction (Figure S6), confirming their critical role as identified in the crystal structure.

### Role of ScdA Monomer and Dimer in Determining Activity

Having established that ScdA is a di-iron nitrite reductase, we next investigated how its oligomeric state influences catalytic activity. In the standard MV assay, the MV cation radical serves as an electron donor but does not effectively break disulfide bonds between ScdA monomers. Consequently, WT ScdA and the S77E variant (analyzed in Figure 5) remain partially oligomeric under our MV-based in vitro conditions. Our SEC analysis showed that over 70% of the as-isolated WT exists as disulfide-linked oligomers, regardless of the presence of MV radicals (Figure S5C). To ensure predominantly dimeric or monomeric forms of ScdA, we conducted MV assays with CF and CF-S77E mutants (Figure 6A), thereby eliminating disulfide-bond formation.

**Figure 6:**
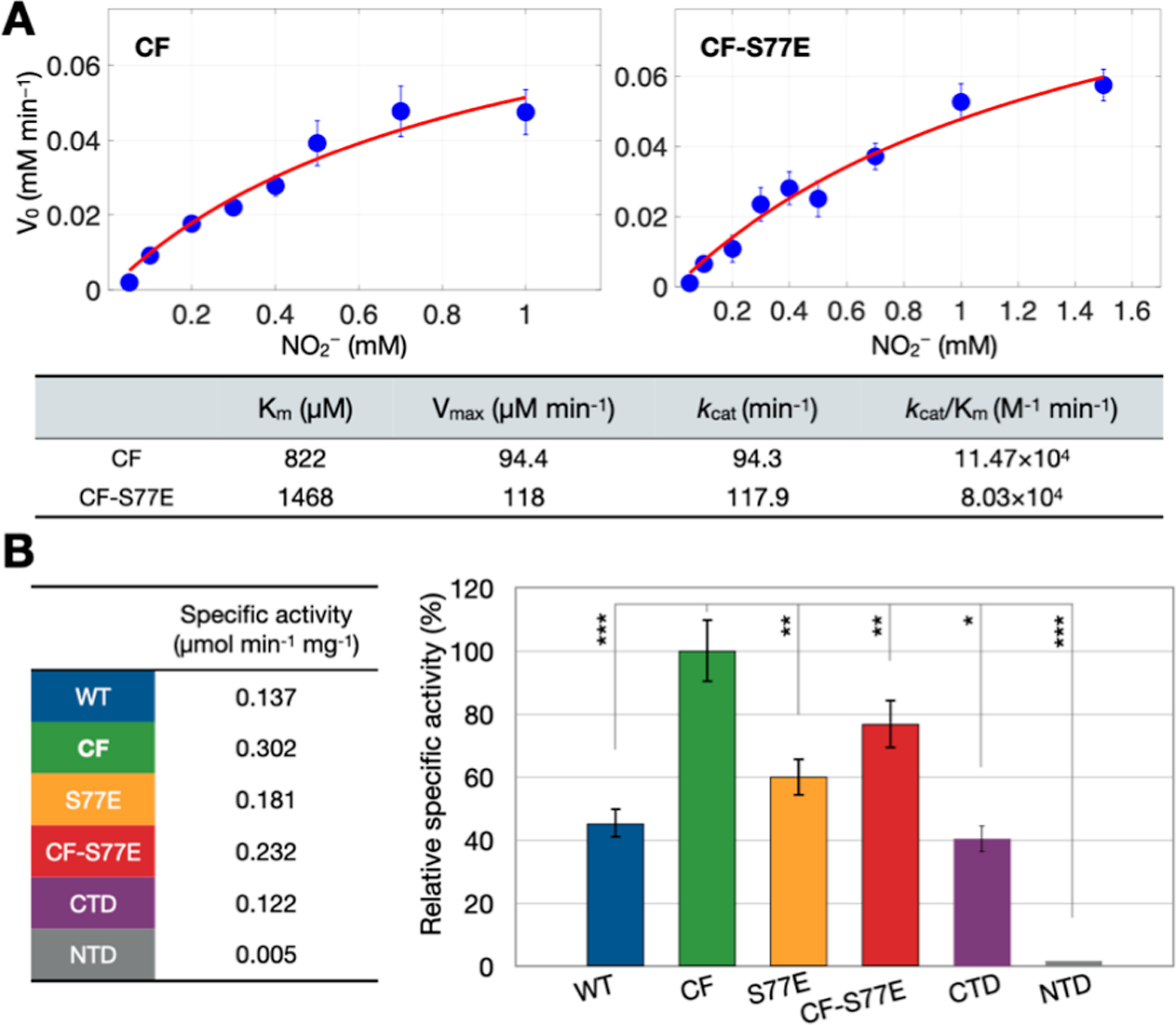
Kinetic Investigations of Full-Length and Truncated ScdA Variants. (A) Michaelis–Menten fits (red lines) of initial reaction rates (*V*_0_) for CF and CF-S77E at pH 7.6, highlighting how eliminating inter-ScdA disulfide-bond formation affects catalytic performance. Error bars represent ±standard deviation. The estimated uncertainty for the reported kinetic parameters is ±5%, based on error propagation from multiple independent measurements (*n* ≥ 3). (B) Relative specific activities of WT, S77E, CF-S77E, CTD-only, and NTD-only variants, normalized to CF (set at 100%), which predominantly adopts a dimeric state. Data shown are for a fixed amount of nitrite (100 μM), and are presented as mean ± standard error (*n* ≥ 3 independent experiments). *p ≤ 5%; **p ≤ 1%; ***p ≤ 0.1%. Note that kinetic parameters were derived with an artificial electron donor and thus reflect intrinsic catalytic potential rather than in-cell turnover.

Notably, the catalytic efficiency (*k*_cat_/*K*_m_) for CF ScdA is ∼2.4-fold higher than that of WT, indicating that the three native cysteines (C30, C31, C191) are not strictly required for nitrite reduction. More importantly, this enhanced efficiency highlights the significance of the dimeric conformation. A similar trend emerges when comparing CF (primarily dimeric) and CF-S77E (primarily monomeric), with CF displaying ∼1.4-fold higher *k*_cat_/*K*_m_ than CF-S77E (Figure 6A).

Interestingly, despite the lower overall catalytic efficiency of CF-S77E, its turnover rate (*k*_cat_) surpasses that of both CF and WT, suggesting that monomeric ScdA may facilitate more rapid substrate turnover once binding occurs. In contrast, CF’s higher *k*_cat_/*K*_m_ reflects better substrate affinity or a more favorable active-site arrangement in the dimeric state. Thus, the oligomeric state appears to influence both substrate binding and conversion rates: while the monomeric form offers faster individual turnover, the dimeric form achieves superior overall efficiency. These findings highlight a subtle interplay between dimerization and catalytic performance.

In addition, we assessed the specific activity (defined as the amount of MV cation radicals oxidized per minute per milligram of protein) of several key ScdA variants at a fixed amount of nitrite (100 μM). These include WT, CF, S77E, CF-S77E, the CTD-only fragment, and the NTD-only fragment (Figure 6B). The NTD-only fragment (monomeric; Figure S5B), which lacks iron, exhibited negligible activity (∼1% of CF’s activity), confirming that it does not itself catalyze nitrite reduction. By contrast, the CTD-only fragment (dimeric; Figure S5B) exhibited ∼40% of the activity seen in the dimeric CF variant, underscoring the essential role of the di-iron center and indicating that the NTD substantially augments maximal catalytic performance. Specifically, the NTD may enhance substrate binding or promote optimal domain arrangement, thus improving the efficiency of electron transfer and product release. Because WT and S77E remain partially oligomeric, their specific activities reached only 45–60% of CF levels. Notably, CF-S77E (monomeric) showed higher activity than S77E, likely reflecting an improved turnover rate at the di-iron site in the absence of disulfide-linked oligomers.

Overall, these findings demonstrate that ScdA is most catalytically effective in a dimeric form and that higher-order oligomerization dampens activity. Moreover, the NTD, though not directly catalytic, bolsters the performance of the CTD, implying that both domains collaborate in optimizing nitrite reduction. This interplay between domain architecture and oligomeric state may help *S. aureus* balance the production of nitric oxide, potentially serving as a regulatory mechanism to avoid excessive NO accumulation.

### ScdA-Mediated Nitrite Reduction Impairs *E. coli* Growth via NO Generation

To evaluate whether the nitrite-to-NO conversion observed in vitro translates into a biologically relevant effect, we examined the impact of ScdA overexpression on *E. coli* cell growth in the presence of nitrite. Cultures were monitored by optical density (OD_600_) over a 10 h incubation period (Figure 7A). Under normal conditions (i.e., without nitrite), cells grew comparably regardless of ScdA overexpression. However, supplementation with 5 mM nitrite led to a pronounced inhibitory effect on the growth of ScdA-overexpressing cultures, whereas control cells without ScdA overexpression were largely unaffected. These results suggest that *E. coli* cells harboring ScdA are more susceptible to nitrite-induced cytotoxicity, presumably due to enhanced NO production. Additional control experiments are provided in the Supporting Information (Figure S7).

**Figure 7:**
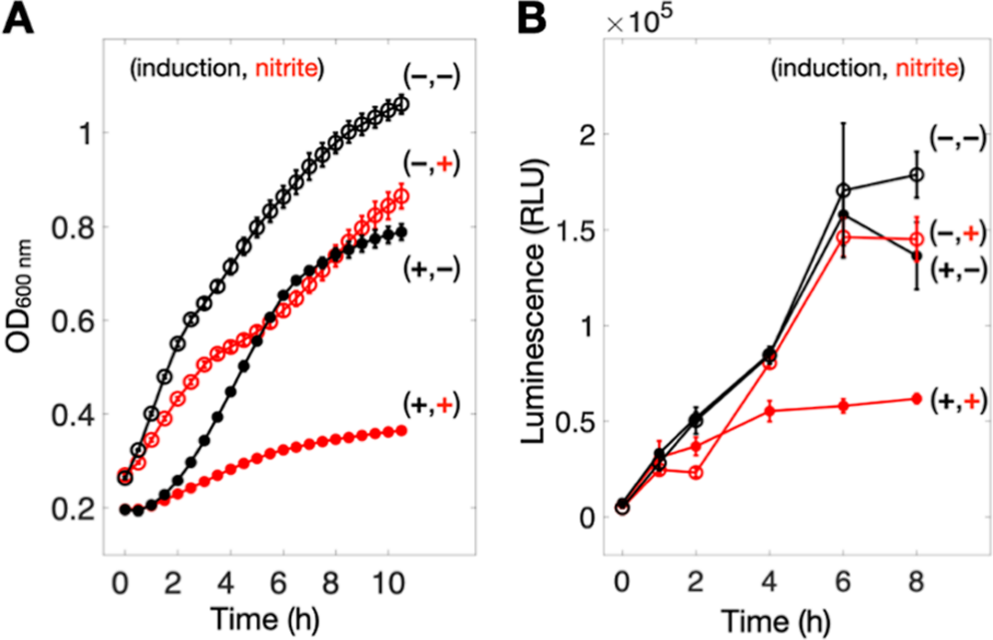
In vivo validation of ScdA-mediated nitrite reduction. (A) Cell growth (OD) and (B) ATP-based luminescence assays used to monitor *E. coli* viability in the presence or absence of 5 mM nitrite, with or without overexpression of WT ScdA. The addition of nitrite has negligible effect on control cells, but markedly inhibits growth and viability in cultures overexpressing ScdA. Data are presented as mean ± standard error (*n* ≥ 3 independent experiments). See also Figure S8 for additional control experiments.

To corroborate these findings, we performed a bioluminescent ATP assay, which quantifies cell viability based on the luciferase reaction driven by intracellular ATP (Figure 7B). Consistent with the OD_600_ measurements, cultures overexpressing ScdA displayed markedly reduced viability in the presence of 5 mM nitrite, whereas cells without ScdA overexpression were largely unaffected. Taken together, these observations strongly indicate that ScdA-mediated nitrite reduction results in elevated intracellular NO levels, ultimately impairing cell growth, a conclusion in line with our in vitro demonstration of ScdA’s ability to generate NO.

Although our study did not employ an *S. aureus* strain lacking scdA gene, the marked growth inhibition observed in *E. coli* overexpressing ScdA strongly suggests that ScdA-catalyzed NO production is substantial under these conditions. Previous reports indicate that *E. coli* typically tolerates nitrite concentrations of up to approximately 60 mM without notable growth inhibition, which is an order of magnitude above the 5 mM used in our experiments.^28^ Thus, the pronounced inhibitory effect we observe likely stems from ScdA-mediated NO generation rather than direct nitrite toxicity. Taken together, these findings indicate that ScdA could represent a critical vulnerability amenable to therapeutic exploitation. In *S. aureus*, it is plausible that ScdA activity is tightly regulated to produce low or localized NO, thereby contributing to physiological or signaling processes while still safeguarding essential metalloenzymes. Further research using *S. aureus* knockout strains and targeted regulatory gene deletions will be needed to clarify how ScdA’s protective and potentially harmful outcomes are balanced in its native host environment.

### Structural Variation in ScdA and Its Implications for Dimerization

To better characterize the structural underpinnings of ScdA function, we compared *S. aureus* ScdA with *E. coli* YtfE (Figure S9A).^29^ Despite a relatively modest sequence identity of 27.5% (66/240) and similarity of 47.5% (114/240), both proteins have a similar two-domain (NTD-linker-CTD) architecture and conserve the core di-iron ligands (four histidine and two glutamate residues). By contrast, the linkers connecting the NTD and CTD differ markedly in length and sequence, with ScdA possessing a longer linker (residues 62–74) than YtfE (residues 60–65). ConSurf analysis^30^ further indicates that these linker residues exhibit high variability across RIC homologues, implying a lower evolutionary pressure to maintain strict conservation in this region (Figure S9B).

To visualize how the linker region contributes to ScdA’s dimeric arrangement, we employed YRB-based color coding, which highlights hydrophobic (yellow) and charged (blue or red) moieties at an atomic level (Figure S9C).^31^ We found that six nonpolar residues (e.g., Pro, Gly, Leu, Phe) in the ScdA linker create a hydrophobic patch that fits into a complementary pocket in the opposing monomer, stabilizing the dimer interface. Additionally, the spatial arrangement of Ser77 (S77) and Gln80 (Q80) enables intermonomer hydrogen bonding. This interplay of hydrophobic contact and hydrogen bonding underscores how a seemingly variable linker can nonetheless facilitate dimerization in ScdA.

### Dimerization and Functional Divergence of ScdA in *S. aureus*

Our results (Figures 5 and 6) indicate that CF ScdA, which predominantly forms dimers, achieves a ∼1.4-fold higher *k*_cat_/*K*_m_ value than CF-S77E (primarily monomeric) and a ∼2.4-fold higher *k*_cat_/*K*_m_ than WT (partially oligomeric under our in vitro conditions). These findings suggest that dimerization is crucial for optimal enzymatic activity. In this context, the intermolecular disulfide bonds and the extended linker in ScdA may have evolved to meet *S. aureus*-specific requirements (such as promoting interactions with regulatory partners, allowing partial compartmentalization of reactive intermediates, or regulating catalytic efficacy by shifting the equilibrium among monomers, dimers, and oligomers) without compromising the di-iron center’s capacity to reduce nitrite to NO. Notably, many bacterial proteins rely on redox-sensitive disulfide bonds to modulate oligomeric states in response to fluctuating oxidative conditions.^32,33^ ScdA may employ a similar mechanism, adjusting disulfide bond formation according to the redox environment and thus tuning the extent of oligomerization to control its nitrite reductase activity. Through such fine-tuning, *S. aureus* can maintain NO production at beneficial levels, mitigating the risk of excessive radical accumulation while preserving the protective and catalytic roles of ScdA.

Additionally, although both *S. aureus* ScdA and *E. coli* YtfE convert nitrite to NO and share a two-domain architecture, their functional properties diverge in several important ways.^11,13,14^ First, ScdA exists as a dimer in *S. aureus*, whereas YtfE remains monomeric in *E. coli*. Second, while we previously developed an NADH-based assay demonstrating that YtfE can reduce NO to N_2_O,^13,14^ we confirmed that ScdA lacks this capability. Third, kinetic analysis shows that YtfE binds nitrite more tightly than the CF variant of ScdA (*K*_m_ = 252 μM vs 822 μM), yet turns it over only about one-third as fast (*k*_cat_ = 34.8 min^–1^ vs 94.3 min^–1^). We compare YtfE with the CF ScdA dimer because dimerization constitutes ScdA’s functional state. Consequently, the two enzymes achieve comparable catalytic efficiencies (*k*_cat_/*K*_m_), highlighting a balance between substrate binding and turnover rate that appears to be optimized differently in each protein. Together, these observations reinforce the lineage-specific adaptations of RIC-family proteins and underscore how each organism may fine-tune oligomerization, substrate affinity, and catalytic turnover to meet its physiological demands.

### NO Management and the Multifaceted Role of ScdA

From a mechanistic standpoint, the in vivo data presented in the present study underscore the potent cytotoxicity of NO. Although *S. aureus* ScdA has previously been implicated in protecting iron–sulfur cluster enzymes from nitrosative and oxidative stress,^5^ our finding that ScdA can also produce NO introduces a seeming paradox. One plausible resolution is that *S. aureus* exerts stringent transcriptional and post-translational control over ScdA, coordinating its activity with additional NO-management pathways (such as spatial compartmentalization or dedicated NO-scavenging enzymes) to avert uncontrolled buildup of the radical. Such regulation would allow the bacterium to harness low or locally restricted NO concentrations for adaptive signaling while preserving iron–sulfur cluster integrity via ScdA’s di-iron site. Notably, endogenous NO production similarly promotes the survival of several human pathogens (e.g., *Bacillus anthracis* and *Moraxella catarrhalis*),^34,35^ implying that pharmacological disruption of ScdA-mediated NO homeostasis may offer a promising anti-MRSA strategy.

In contrast, overexpressing *S. aureus* ScdA in *E. coli* floods the cell with NO beyond what the host’s native detoxification systems can counter, causing marked growth inhibition in nitrite-rich media. The long-standing view that ScdA protects iron–sulfur clusters therefore captures only part of its function. Like several other bacterial enzymes, ScdA likely plays multiple roles in nitrosylative-stress management—serving at times as an NO sink and at other times as an NO source. Flavodiiron proteins, for example, typically detoxify NO but can also reduce O_2_ or even generate NO depending on environmental or genetic context.^36,37^ Likewise, certain bacterial cytochrome *c* oxidases, though optimized for the four-electron reduction of O_2_ to H_2_O, catalyze a slower side reaction in which two NO molecules are reduced to N_2_O at the same heme-Cu site, underscoring their bifunctionality.^38,39^ Collectively, such examples show that an enzyme’s apparent benefit or toxicity is dictated less by its intrinsic chemistry than by the cellular regulatory and metabolic network in which it is embedded.

In summary, this study used an integrative approach combining X-ray crystallography, NMR spectroscopy, AlphaFold predictions, and DEER technique to determine the complete, dimeric structure of *S. aureus* ScdA. The high-resolution X-ray data defined the CTD as a four-helix bundle containing the di-iron center, while NMR spectroscopy resolved the NTD, which was uncharacterized in the crystallographic data. AlphaFold modeling of the full-length ScdA aligned well with both X-ray and NMR structures, and DEER experiments corroborated the solution-state dimer interface and conformation. Functionally, kinetic assays and EPR reveal that ScdA reduces nitrite to NO, with in vivo overexpression in *E. coli* impairing growth under nitrite-rich conditions, likely due to NO toxicity. Comparative analyses of cysteine-free, interface, and diiron-coordinating mutants underscore how key residues and oligomeric states shape catalytic efficiency. Collectively, these findings highlight ScdA as a di-iron protein capable of generating NO, thereby broadening the known activities of the RIC family and illustrating how a protein long associated with iron-sulfur cluster maintenance can also produce a cytotoxic radical. Our results extend our understanding of bacterial stress responses and lay the groundwork for novel research into NO regulatory networks. Ultimately, these insights could guide the design of strategies to manipulate NO-based mechanisms in antibiotic-resistant pathogens, including *S. aureus*.

## Materials and Methods

### Protein Sample Preparation

The full-length *S. aureus* scdA gene was inserted into the NcoI/EcoRI sites of the pET-28a expression vector (New England Biolabs). This vector adds an N-terminal six-His tag followed by a thrombin-cleavage site, yielding purified ScdA at ∼27 kDa with the tag (Figure 1A) and ∼25 kDa after tag removal (Figure S1B). A C-terminal domain-only (CTD, residues 64–224) variant was similarly cloned into pET-28a using the same procedure. The wild-type (WT) ScdA construct served as the template for site-directed mutagenesis. Since WT ScdA contains three native cysteine residues (C30, C31, and C191), a cysteine-free (CF) construct (C30A/C31A/C191A) was generated for spin-labeling experiments. An N-terminal domain–only (NTD) variant was obtained by introducing a stop codon at T64 (T64stop). Mutations were introduced using the QuikChange Site-Directed Mutagenesis Kit (Stratagene) with complementary primers and verified by DNA sequencing. All plasmids were transformed into *E. coli* BL21­(DE3) cells (Agilent) for expression.

Unless specified otherwise, all recombinant proteins included an N-terminal His_6_-tag. Transformants were first grown in 50 mL Luria–Bertani (LB) medium containing 40 μg/mL kanamycin overnight at 37 °C. The following day, 50 mL of overnight culture was inoculated into 500 mL fresh LB. At OD_600_ ≈ 1.0, 0.2 mM FeCl_3_ and 0.4 mM IPTG were added, and cultures were grown at 30 °C for 6 h. Cells were harvested by centrifugation and stored at −80 °C until further processing.

Cell pellets were resuspended in lysis buffer (20 mM Tris, 150 mM NaCl, pH 7.6) supplemented with 0.6 mM PMSF and lysed on ice by sonication for 10 min. Lysates were clarified by centrifugation at 23,000*g* for 50 min, and the supernatant was passed through a 0.45 μm filter prior to loading onto a 5 mL HisTrap HP nickel-affinity column (GE Healthcare). The column was washed with 50 mL wash buffer (20 mM Tris, 150 mM NaCl, 45 mM imidazole, pH 7.6) and eluted with 25 mL elution buffer (20 mM Tris, 150 mM NaCl, 300 mM imidazole, pH 7.6). Imidazole was removed using a PD-10 desalting column (GE Healthcare) pre-equilibrated with storage buffer (20 mM Tris, 150 mM NaCl, 10% v/v glycerol, pH 7.6). Purified proteins were verified on a Superdex 75 Increase 10/300 GL column (Cytiva) using 20 mM Tris, 150 mM NaCl (pH 7.6) as the running buffer, and by SDS-PAGE with Coomassie blue staining. For the SDS-PAGE analysis, ScdA (0.02 mM, 10 μL) was mixed with 2.5 μL 5 × SDS loading buffer (250 mM Tris–HCl, 12.5% SDS, 50% glycerol, 5 mM EDTA, 50 μg/mL bromophenol blue, 500 mM DTT) and denatured at 95 °C for 6 min. The samples were resolved on a 15% SDS–PAGE gel and stained with Coomassie blue to visualize the protein band. Protein concentrations were measured by UV–vis spectroscopy at 280 nm, using extinction coefficients of 40,800 M^–1^ cm^–1^ for full-length ScdA, 37,820 M^–1^ cm^–1^ for the CTD, and 3105 M^–1^ cm^–1^ for the NTD.

Purified cysteine variants were incubated overnight at 4 °C in the dark with a 40-fold molar excess of MTSSL (1-oxyl-2,2,5,5-tetramethylpyrroline-3-methyl methanethiosulfonate; Enzo Life Sciences). Unreacted spin label was removed by exchanging into storage buffer.

### NMR Sample Preparation

Isotopically labeled NTD-only construct (C30A/C31A) was expressed in *E. coli* grown initially in 50 mL LB containing 40 μg/mL kanamycin at 37 °C overnight. Cells (OD_600_ = 3–5) were pelleted, resuspended in 500 mL M9+ medium (containing 0.01% thiamine, 40 μg/mL kanamycin, 1 g/L ^15^N–ammonium chloride, and 2 g/L ^13^C–glucose), and supplemented with 0.2 mM FeCl_3_. Protein expression was induced by 0.4 mM IPTG at 37 °C for 2 h, followed by 25 °C for 20 h. Cells were harvested, and the NTD protein was purified as described above. The His_6_-tag was cleaved using TEV protease. Purity was confirmed by SDS-PAGE and a Superdex 75 Increase 10/300 GL column.

### NMR Experiments

Spectra were recorded at 25 °C on Bruker AVANCE 600 and 850 MHz spectrometers. Backbone resonance assignments were obtained from HNCA, HN­(CO)­CA, HNCO, HNCACB, and HN­(CO)­CACB data sets. Sidechain assignments were derived from HBHA­(CO)­NH, H­(CC)­(CO)­NH, CC­(CO)­NH, and HCCH-TOCSY. Nonuniform sampling (NUS) was used to improve spectral quality in 3D experiments. Data were processed with NMRPipe and analyzed in NMRFAM-SPARKY.^40,41^ The assigned ^1^H, ^15^N, and ^13^C chemical shifts have been deposited in the BioMagResBank (accession code 52893). Secondary structures were identified by comparing experimental Cα and Cβ chemical shifts with random coil values (ΔCα – ΔCβ). TALOS-N calculations confirmed four α-helices.^42^ Backbone chemical shifts were also used in CS-Rosetta to generate 3D structural models.^43^

### X-ray Crystallography: Sample Preparation

A tag-free variant of CF ScdA (referred to here as “tagless ScdA”) was generated by removing the nucleotide region encoding the six-histidine and TEV-cleavage site from pET_His-ScdA CF. The deletion was achieved using the KLD enzyme mix (New England Biolabs) with primers 5′-ATGATCAACAAGAACGAC-3′ and 5′-GGTATATCTCCTTCTTAAAG-3′, yielding an open reading frame identical to the native *S. aureus* sequence. The expression protocol matched that of the His-tagged ScdA. *E. coli* cells were resuspended in 20 mM Tris–HCl (pH 7.5) containing 1 mM PMSF, 10 μg/mL DNase I, and 1 mM MgCl_2_, then disrupted using a French press. After incubation with 10 mM CaCl_2_ for 30 min to precipitate unwanted proteins, the lysate was centrifuged at 11,000 × *g* for 1 h. The supernatant was loaded onto a self-packed HiLoad 16/10 Q Sepharose Fast Flow column (Cytiva) and washed sequentially with 20 mM Tris–HCl (pH 7.5), 150 mM NaCl, and 20 mM Tris–HCl (pH 7.5), 200 mM NaCl. A linear gradient from 100 mM to 1 M NaCl in 20 mM Tris–HCl (pH 7.5) was applied using an ÄKTA pure system (Cytiva). The eluted protein was further purified by size-exclusion chromatography on an ENrich SEC 650 10/300 column (Bio-Rad). Fractions containing tagless ScdA were pooled and concentrated to ∼10 mg/mL in 20 mM Tris–HCl (pH 7.5) using a 3 kDa MWCO concentrator (Pall).

### X-ray Crystallography: Data Collection and Structure Refinement

Crystallization trials were set up with tagless ScdA (10 mg/mL) and a crystallization reagent comprising 0.2 M CaCl_2_, 0.1 M MES (pH 6.0), 20% (w/v) PEG 6000, and 0.1 M β-NAD, mixed at a 1:1 volume ratio. Initial crystals were grown at 24 °C by sitting-drop vapor diffusion. Crystal quality was improved by microseeding with serially diluted seeds in the same crystallization mixture at 24 °C. Crystals were transferred to the mother liquor supplemented with 25% ethylene glycol as a cryoprotectant and flash-cooled in liquid nitrogen. Diffraction data were collected at the TPS synchrotron BL05A beamline (NSRRC, Taiwan) using an EIGER2 × 9 M detector. Key data collection and refinement statistics are listed in Table S1. The native wavelength was 0.99987 Å. HKL2000 ^44^ was used for indexing and integration, revealing a *P*2_1_2_1_2_1_ space group with unit cell dimensions *a* = 69.54 Å, *b* = 92.49 Å, *c* = 110.89 Å, and α = β = γ = 90°. Phasing was performed by Phaser (PHENIX suite) using the AlphaFold-predicted ScdA dimer as a search model. Manual model building and adjustments were carried out in COOT.^45^ Low electron density in the N-terminal domain prevented confident placement of the first 66 residues, so they were excluded. Final refinement proceeded with phenix.refine^46^ until *R*_free_ ceased to decrease, and the structure was deposited in the PDB (9J47, 9W8G).

### UV–Vis Observation of Iron–Nitrosyl Complex Formation

To assess ScdA-mediated nitrite reduction, ScdA (0.2 mM) was subjected to three freeze–pump–thaw cycles under anaerobic conditions. The reduced form of ScdA was prepared by incubating with a 100-fold molar excess of DTT for 1 h. After adding 2 mM NaNO_2_, the characteristic 397 nm iron-nitrosyl absorption peak was recorded by a Hitachi U-3900 spectrometer, indicating NO generation.

### Nitrite Reductase Assay

Methyl viologen (MV) was reduced according to a previously reported photochemical protocol, with all steps carried out under strictly anaerobic conditions.^47^ Briefly, 4 mg MV was dissolved in 1 mL ethanol and irradiated at 365 nm until the solution acquired a deep blue color. The solvent was then evaporated, and the remaining material was resuspended in the assay buffer, yielding reduced MV.

For the enzymatic measurements, 116 μM reduced MV and 1 μM ScdA were mixed in an anaerobic cuvette, and nitrite (NaNO_2_) was injected (final concentration: 3 μL of 100 μM stock).^11^ Absorbance at 600 nm (extinction coefficient 13.70 mM^–1^ cm^–1^) was recorded up to 1200 s to follow MV oxidation. Initial reaction rates (*V*_0_) were determined by linear fitting of the early time points and subsequently plotted against varying nitrite concentrations. The resulting data were analyzed with two methods: nonlinear regression to the Michaelis–Menten equation and linear transformation in the form of a Lineweaver–Burk plot. Both analyses yielded comparable kinetic parameters (*K*_m_, *V*_max_, *k*_cat_) within a ±5% margin of error, confirming the robustness of the fit. This dual analytical approach underscores the reliability of the kinetic constants, ensuring accurate quantification of ScdA’s nitrite reductase activity.

### Continuous Wave (CW) ESR Measurements

For CW-ESR, samples were buffer-exchanged into deuterated buffer with 30% (w/w) d_8_-glycerol as a cryoprotectant. Final protein concentrations were ∼0.2 mM, loaded into sealed 20 μL glass capillaries. Measurements were conducted at 10 K on a Bruker ELEXSYS 580 equipped with an X-band microwave bridge and an ER 4122 SHQE cavity. Acquisition parameters included: 9.7 GHz microwave frequency, 100 kHz modulation, 1 G modulation amplitude, and 1.5 mW incident microwave power.

### Pulse ESR Measurements and Data Analysis

For DEER experiments, spin-labeled samples were similarly buffer-exchanged into deuterated buffer containing 30% (w/w) d_8_-glycerol. Protein concentration (∼0.2 mM) was loaded into 3 mm quartz tubes and frozen in liquid nitrogen. DEER experiments were conducted on a Bruker ELEXSYS E580–400 X-band cw/pulse spectrometer with a split-ring resonator (EN4118X-MS3) equipped with a cryogenic ultralow-noise microwave amplifier and a helium gas flow system (4118CF and 4112HV) at 80 K.^48,49^ We used a four-pulse constant-time DEER sequence (two-step phase cycling), with a π/2 pulse of 16 ns, a π pulse of 32 ns, and a pump–probe frequency offset of 65 MHz.^18,50^ The pump pulse was positioned at the maximum of the echo-detected field-swept spectrum. All pulses were amplified using a pulsed traveling wave tube (TWT) amplifier (E580–1030), with data accumulation times ranging from 30 to 60 min. The DeerLab software package was used to remove background signals and apply Tikhonov regularization to derive model-free distance distributions.^22,23,51^ Further model-based analyses (sum of Gaussians) and error estimations were performed with DD software (version 7) to provide an independent check.^52,53^ Both approaches yielded consistent distance distributions (Figure 2B). Distance distribution simulations based on the AF-model were conducted using the rotamer ensemble models implemented in the chiLife software.^24^ These simulations account for side-chain flexibility and conformational sampling, providing a statistically robust prediction of interspin distances.

### In-Cell Viability Assays

Two complementary assays, OD_600_ monitoring and ATP-based luminescence (BacTiter-Glo), were used to measure *E. coli* viability. Transformants carrying ScdA in pET-28a were grown in 50 mL LB medium (40 μg/mL kanamycin) overnight at 37 °C. Cells were pelleted and resuspended in 50 mL LB supplemented with 0.2 mM FeCl_3_, then incubated at 37 °C for 1 h. Protein expression was induced by adding 0.4 mM IPTG at 30 °C for 6 h. Cultures were adjusted to OD_600_ = 0.1 in fresh LB and incubated at 37 °C for 1 h to reach a stable state. For OD_600_-based measurements, 200 μL aliquots were dispensed into a 96-well transparent plate (GeneDireX) with or without 5 mM nitrite and monitored at 30 °C for 10 h using a Synergy H1 Microplate Reader (BioTek).

For the luminescence assay, 3 mL cultures adjusted to OD_600_ = 0.1 were also stabilized at 37 °C for 1 h. After treatment with or without 5 mM nitrite for 8 h, 100 μL of each culture was mixed with 100 μL BacTiter-Glo reagent in a black 96-well plate (Thermo Scientific) to measure ATP-dependent luminescence.

### Statistical Analysis and Reproducibility

All statistical analyses used two-tailed unpaired Student’s *t*-tests. Significance levels were denoted by asterisks (ns > 5%; *p ≤ 5%; **p ≤ 1%; ***p ≤ 0.1%.), as shown in Figure 6B. Unless otherwise noted, data are presented as the mean ± standard error (SE).

## Supplementary Material



## Notes

Figures S1–S4 show experimental data of the X-ray crystal, sequence alignment, NMR spectroscopy, and DEER measurements. Figure S5 presents Supporting Information from UV–vis and SEC measurements. Figure S6 shows the UV–vis and CW EPR data for the MV assays. Figure S7 presents the iron content measurements. Figure S8 shows additional control experiments for the cell viability assay. Figure S9 presents the analysis of the dimer interface. Table S1 presents the X-ray collection and refinement statistics (PDF)
