# Supplementary material for: Structure and Nitrite Reductase Activity of the Di-iron Protein ScdA in Staphylococcus aureus
Source: J Am Chem Soc. 2025 Aug 22;147(35):31558–69. doi: 10.1021/jacs.5c05573 (PMC12412097; doi:10.1021/jacs.5c05573)
Supplement: Supplementary file 1 [file ja5c05573_si_001.pdf]

Supplementary Materials for

Structure and Nitrite Reductase Activity of the Di-iron Protein ScdA in *Staphylococcus aureus*

Hung-Ying Chen,[1] Ruei-Fong Tsai,[1] Yi-Shan Lu,[1] Yang-Chun Cheng,[1] Hsiang-Yuan Fan-Chiang,[1] Chu-Ya Wu,[2] Feng-Chun Lo,[1,2] Hsuan-Wei Kuo,[2] Wei-Kai Yang,[3] Wan-Yi Liao,[3] Nien-Jen Hu,[3]* Shih-Che Sue,[2]* Yun-Wei Chiang[1]*

[1]Department of Chemistry, National Tsing Hua University, Hsinchu 300-044, Taiwan

[2]Institute of Bioinformatics and Structural Biology, National Tsing Hua University, Hsinchu 300-044, Taiwan

[3]Graduate Institute of Biochemistry, National Chung Hsing University, Taichung 402-202, Taiwan

*Correspondence emails:

njhu@nchu.edu.tw

scsue@life.nthu.edu.tw

ywchiang@mx.nthu.edu.tw

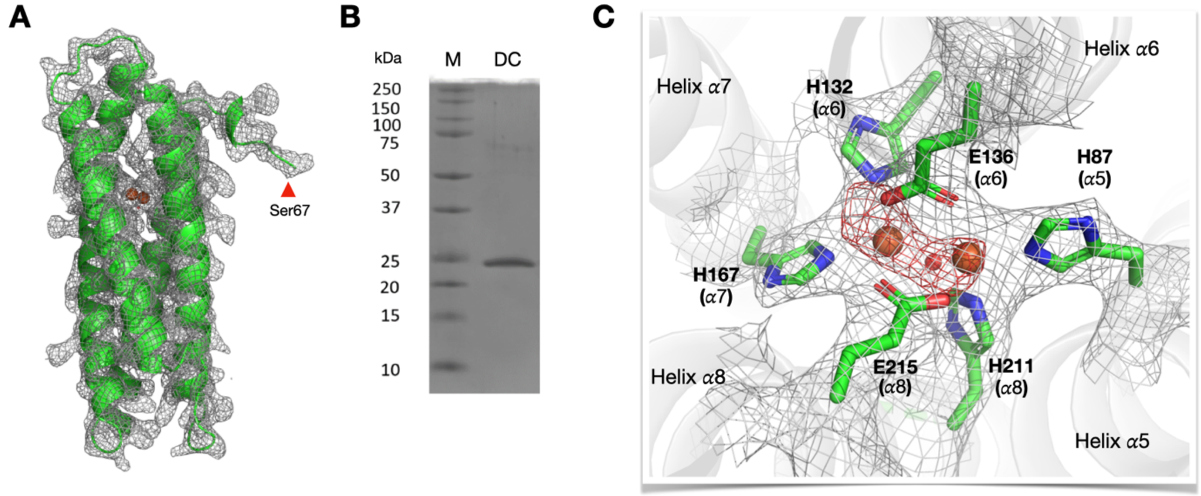

Figure S1. Crystal Structure of the CF ScdA.

(**A**) A single protomer from the dimeric ScdA structure is shown as a green cartoon. The $2F_o$–F_c electron density map is rendered as a gray mesh and contoured at 1.0 σ. Residues from the C-terminus down to Ser67 were confidently traced and built, whereas the N-terminal segment (residues 1–66) lacked well-defined density and was therefore excluded from the final model.

(**B**) SDS–PAGE analysis of dissolved CF ScdA crystals. After harvesting, crystals were repeatedly back-washed in mother liquor devoid of protein, then dissolved for electrophoresis. The gel lane labeled "M" indicates the protein marker, and "DC" denotes the dissolved-crystal sample. The SDS-PAGE analysis of the dissolved ScdA crystals indicated an molecular weight of the intact protein (i.e., His-tag-cleaved ScdA).

(**C**) Close-up view of the di-iron center within the C-terminal domain. Four helices (α5–α8) are shown as cartoons, and side chains for the iron-coordinating residues are drawn as sticks. The two iron atoms are depicted as brown spheres, bridged by a μ-oxo group shown as a red sphere. This arrangement underscores the hemerythrin-like architecture responsible for ScdA's catalytic function. $2F_o$-F_c map is shown in grey mesh and anomalous difference map of iron is shown in red mesh, contoured at 1.4 σ and 3.2 σ, respectively.

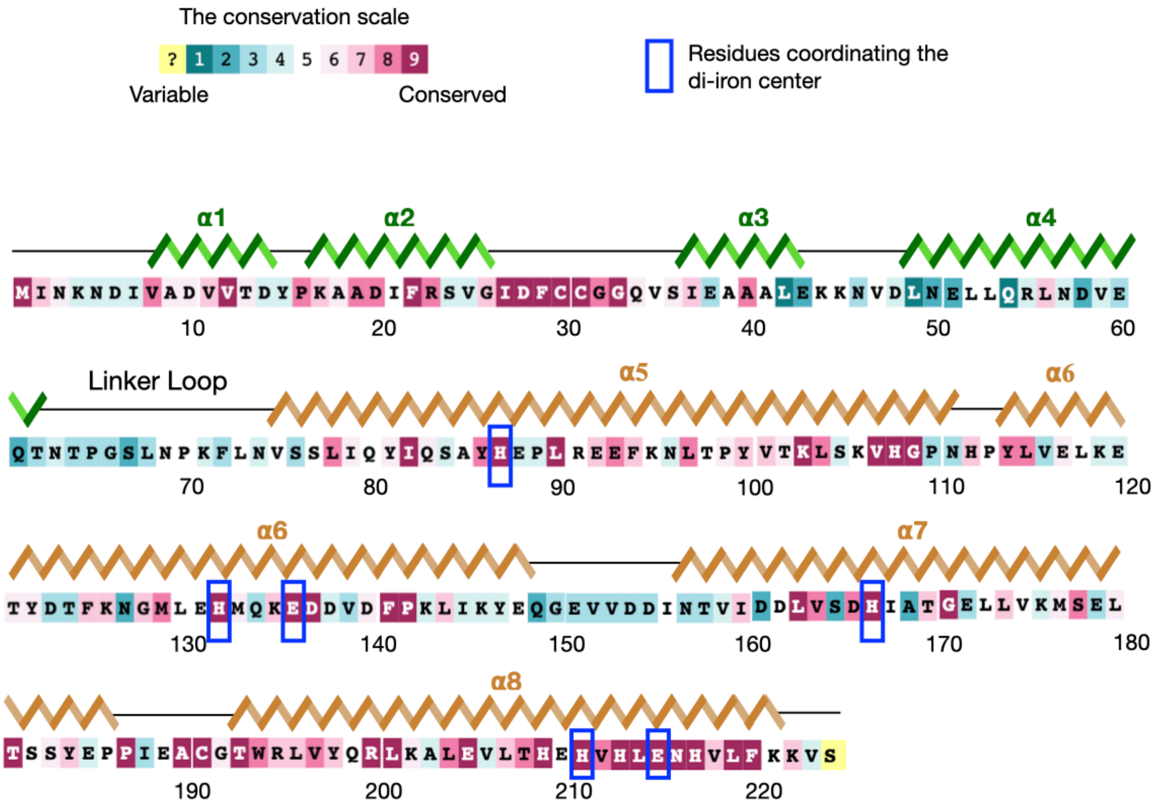

Figure S2. Evolutionary Conservation Analysis of ScdA.

The color scale on the top depicts the degree of conservation assigned by ConSurf. Notably, the six residues (H87, H132, E136, H167, H211, and E215, highlighted by blue boxes) that coordinate the di-iron center exhibit high conservation, consistent with their crucial catalytic role. By contrast, helix α4 at the C-terminus of the NTD and the linker loop (residues 62–74) connecting the NTD and CTD display very low sequence conservation. Although helix α4 of the NTD is weakly conserved, helices α1–α3 are strongly conserved, underscoring the functional importance of the NTD. This conservation pattern mirrors our functional data (Figure 6), which show that both the NTD and CTD are required for efficient ScdA-catalyzed nitrite reduction.

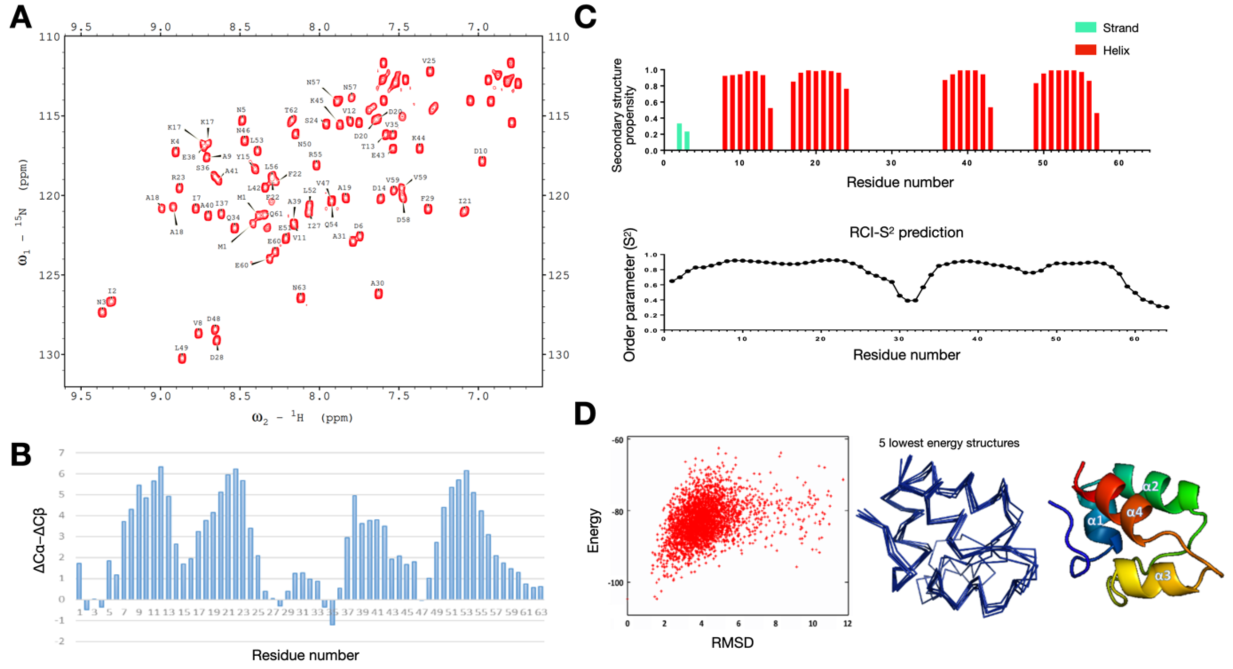

Figure S3. NMR Spectroscopic Analysis of the ScdA NTD (Residues 1-64).

(A) ^{15}N-HSQC spectrum. The spectrum of the NTD exhibits well-dispersed resonances, indicative of a stably folded domain. To complete the backbone assignment prior to degradation, we employed non-uniformly sampled (NUS) three-dimensional NMR experiments (HNCA, HN(CO)CA, HNCO, HNCACB, and HN(CO)CACB). This strategy enabled us to assign 62 out of 63 non-proline residues (98% completeness). Residue T64 remained unassigned, likely due to loop terminus heterogeneity. Additionally, minor peaks for M1, K17, A18, D20, F22, N57, V59, and E60 were detected, suggesting the presence of a minor conformational state. After establishing the backbone assignments, sidechain resonances were assigned to over 90% completeness, leaving only a few aromatic and lysine sidechain signals unresolved.

(B) Backbone chemical shifts (ΔCα–ΔCβ) and secondary structure. Shifts of ΔCα–ΔCβ > 0 indicate α-helical content, whereas values < 0 suggest β-sheet structure. The resulting profile identifies four α-helices in the NTD, corroborated by TALOS-N predictions. Specifically, helix α1 spans residues V8–D14, helix α2 spans residues P16–V25, helix α3 spans residues S36–K44, and helix α4 spans residues L49–D58.

(C) Secondary structure and order parameters. In agreement with the ΔCα–ΔCβ analysis, TALOS-N calculations confirm four helices in the NTD. The loop between helices 2 and 3 (G26–V35) displays notably low order parameter (S^2) values, indicating greater flexibility.

(D) CS-Rosetta 3D-structure prediction. Using backbone chemical shifts as input, CS-Rosetta trimmed the disordered C-terminus and generated 3,000 structural models for residues 1–58. Of these, the five lowest-energy conformations converged with Cα RMSD values of 0.90–1.40 Å (mean 1.09 ± 0.19 Å), demonstrating high structural consistency. Each α-helix is amphipathic, with hydrophobic side chains facing inward to form a compact core. F22 anchors the hydrophobic center, and the domain appears to comprise two subdomains (helices α1– α2 and helices α3–α4) connected by a flexible loop. Helices 2 and 4 pack antiparallel via hydrophobic contacts. Notably, minor-conformation peaks primarily occur in helix α2 (K17, A18, D20, F22) and the C-terminal loop (N57, V59, E60), where a cross-interacting salt bridge is observed between K17 (helix α2) and D58 (terminal loop).

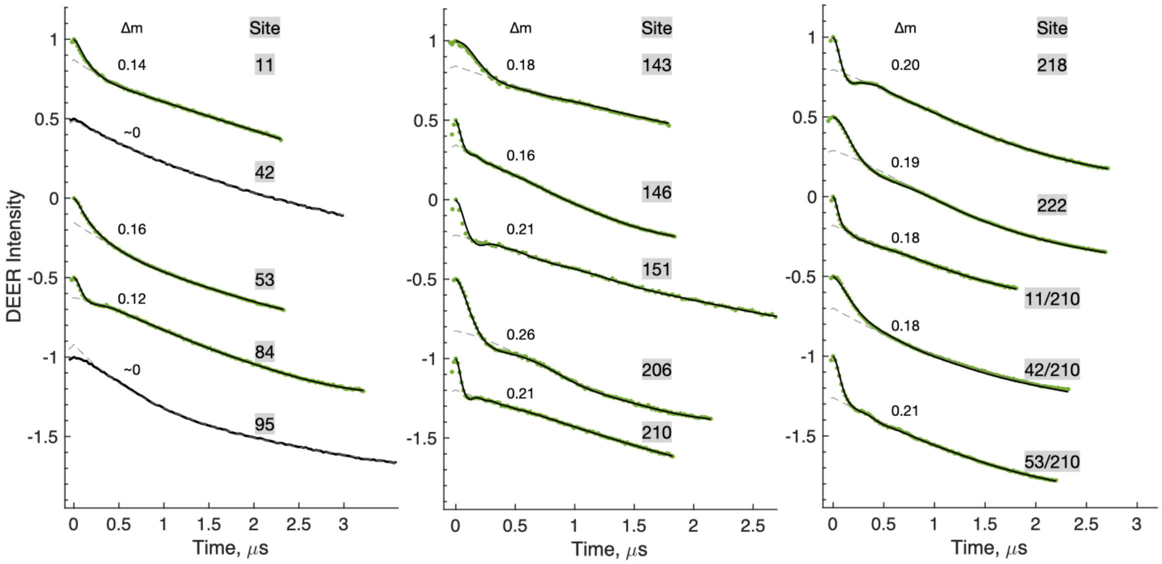

Figure S4. DEER Experimental Data and Analysis.

Green dots indicate the raw DEER time-domain data, labeled by a single residue number for CF dimers (one spin-labeled site per monomer), or by two residue numbers for CF-S77E monomeric variants (two labels on the same polypeptide). Modulation depths (Δm) of the experimental DEER traces are given in the plots. Tikhonov- and Gaussian-based analyses were performed in DeerLab to generate the distance distributions displayed in the main text (Fig. 2B). Gray lines represent the background fit, while black lines show the simulated fit to the experimental data. For ScdA spin-labeled at sites 42 or 95, the dipolar oscillation proved too weak to yield reliable distance distributions. The two variants produced weak dipolar signals suggestive of inter-spin distances exceeding 6 nm, which lies beyond the detection limit of our pulse X-band EPR spectrometer. These findings reinforce the conclusion that the inter-spin distances at those positions exceed the detection range of the current DEER setup.

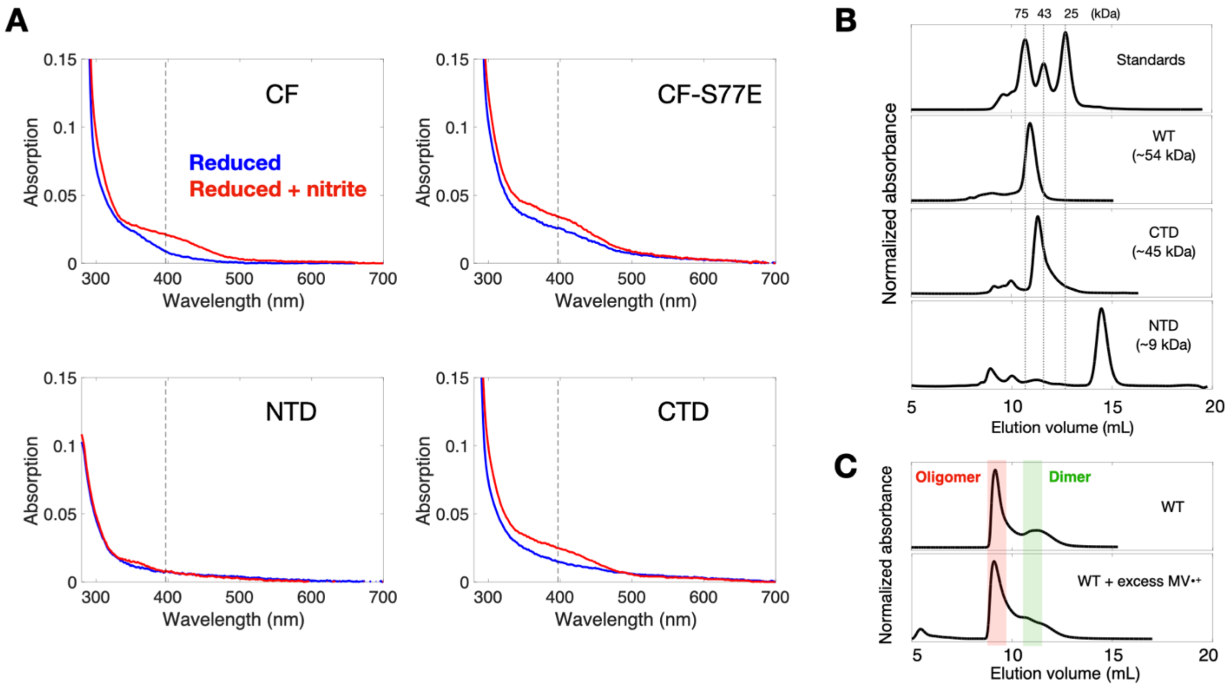

Figure S5. UV–Vis Characterization and SEC Profiles of ScdA Variants.

(**A**) Shown are UV–Vis spectra of CF, CF-S77E, NTD-only, and CTD-only variants following reduction with DTT (blue) and subsequent treatment with excess nitrite (red). The emergence of the 397 nm band upon nitrite addition indicates iron-nitrosyl complex formation in the reduced protein. The data support that CF, CF-S77E, and CTD-only variants can facilitate nitrite conversion to NO in the presence of a reducing agent.

(**B**) SEC profiles of the CTD-only and NTD-only fragments. DTT-reduced WT ScdA is plotted as a reference to indicate where an ScdA dimer elutes in the SEC. The results indicate that the CTD-only fragment predominantly forms a dimer (~45 kDa), whereas the NTD-only fragment remains primarily monomeric (~9 kDa). Standard markers are also provided in the plot.

(**C**) SEC profiles of WT ScdA before and after incubation with excess MV cation radicals (WT:MV$^{\cdot+}$ 1:100). These data indicate that while the MV radical acts as an electron donor in the standard MV assay, it does not effectively disrupt disulfide bonds among ScdA monomers. As a result, both WT ScdA and the S77E variant remain partially oligomeric under our MV-based *in vitro* conditions (Fig. 5).

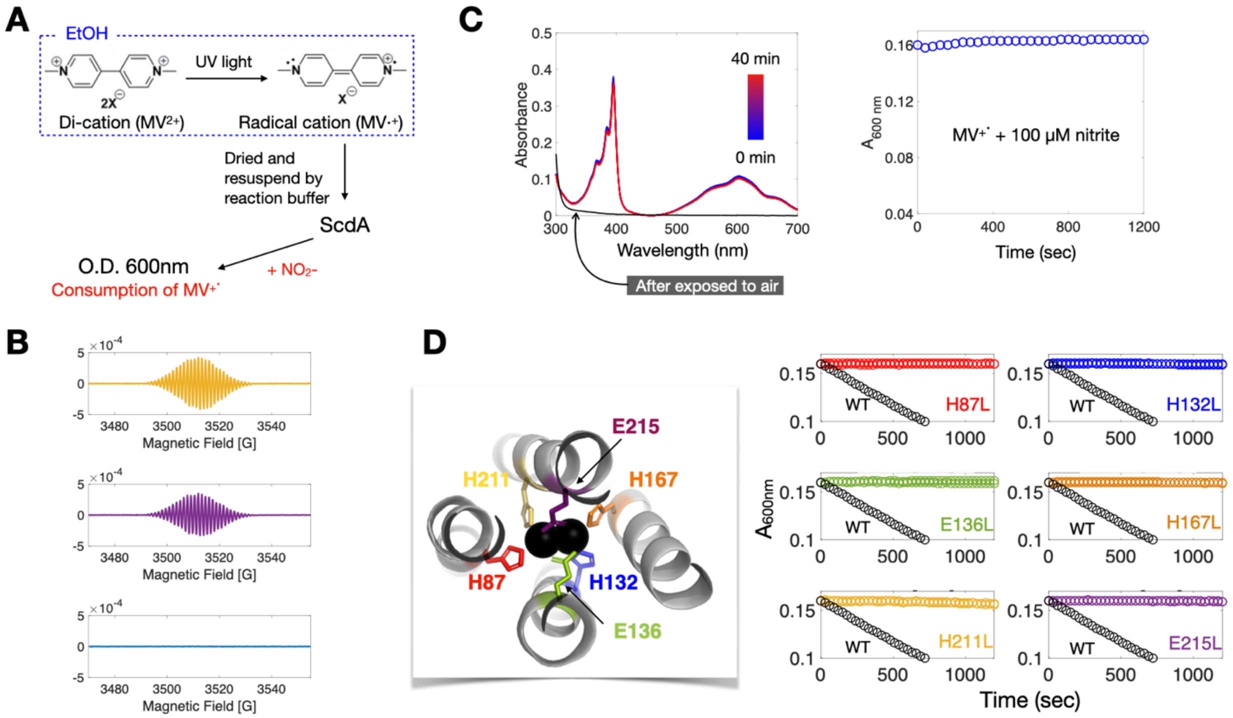

Figure S6. Supplementary Data for the Methyl Viologen (MV) Assay.

(**A**) Schematic illustrating the generation of reduced MV cation radicals ($MV^{\cdot+}$). Briefly, MV dissolved in ethanol is exposed to 365 nm UV light until it acquires a deep blue color. After evaporation of the solvent, the residue is resuspended in reaction buffer to obtain $MV^{\cdot+}$. Under anaerobic conditions, $MV^{\cdot+}$ and ScdA are combined, and the reaction is initiated by injecting $NaNO_2$. Absorbance at 600 nm is monitored to track MV oxidation over time.

(**B**) Continuous-wave (CW) EPR spectra recorded at 300 K for (yellow) 116 µM $MV^{\cdot+}$ alone, (purple) 116 µM $MV^{\cdot+}$ with 1 µM ScdA, and (blue) 116 µM $MV^{\cdot+}$ with 1 µM ScdA plus 100 µM nitrite after 20 minutes. Samples were prepared anaerobically and flame-sealed prior to EPR measurements. The data confirm that $MV^{\cdot+}$ remains stable in the presence of ScdA alone, and is oxidized only upon addition of nitrite.

(**C**) UV–Vis spectra of an anaerobic mixture containing $MV^{\cdot+}$ and nitrite in the absence of ScdA, recorded at 600 nm for 40 minutes. The negligible change in absorbance confirms that $MV^{\cdot+}$ is not consumed by nitrite under these conditions.

(**D**) MV-based assay results showing that substituting any one of the six di-iron–coordinating residues (H87, H132, E136, H167, H211, E215) abolishes nitrite reduction, reinforcing the importance of these residues identified in the crystal structure.

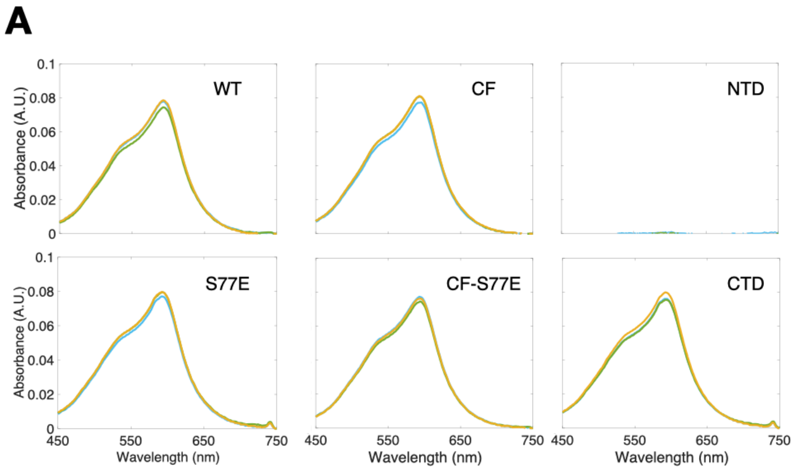

B

	Iron content (Number of iron per monomer)
**WT**	1.81±0.04
**CF**	1.86±0.04
**S77E**	1.85±0.03
**CF_S77E**	1.79±0.03
**NTD**	0.05±0.02
**CTD**	1.82±0.04

Figure S7. Iron Stoichiometry of ScdA Variants Determined by the TPTZ Assay.

(A) UV–visible spectra (450–750 nm) recorded after complexation of acid-liberated metal ions with excess 2,4,6-tripyridyl-s-triazine (TPTZ). Shown are three independent preparations of each sample, plotted in blue, green, and yellow; the near-perfect overlap of the traces demonstrates the excellent reproducibility of iron loading across batches. The characteristic Fe^{2+}–TPTZ charge-transfer band at 595 nm is present for all constructs except the isolated NTD. The assay followed established protocols:[13] as-isolated protein (8 nmol) was denatured in 75 µL 3 M HCl at 60 °C for 1 h, cooled, mixed with 100 µL 1 mM TPTZ and 10 µL 10 mM sodium dithionite, adjusted to pH ≈ 4 with 1 M sodium acetate, and brought to 300 µL total volume. Fe^{2+}–TPTZ was quantified from its 595 nm absorbance using an $FeSO_4$ standard curve.

(B) Iron content expressed as Fe atoms per monomer (mean ± SD, n = 3). Wild-type (WT), cysteine-free (CF), S77E, CF-S77E, and the CTD contain 1.8–1.9 Fe per subunit, whereas the NTD is metal-free. These results indicate that removal of all cysteines or disruption of the dimer interface does not affect the di-iron centre and confirm that metal binding resides within the CTD.

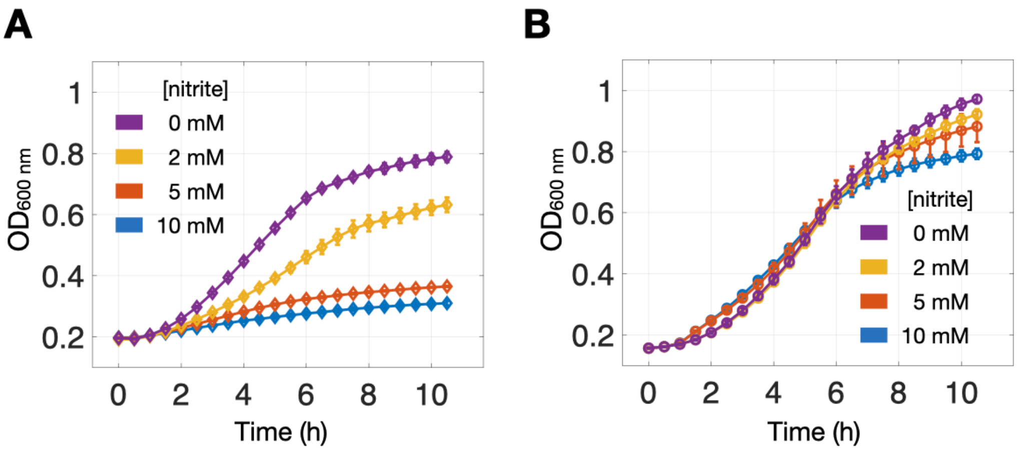

Figure S8. Additional Control Experiments for the Cell Viability Assay.

(**A**) Effect of overexpressing WT ScdA on *E. coli* growth in the presence of increasing nitrite concentrations (0–10 mM). Cell growth, monitored by OD_{600}, remains unaffected in the absence of nitrite but is increasingly inhibited as the nitrite concentration rises. Growth is reduced by approximately 20% at 2 mM nitrite (after 10-h incubation) and is markedly inhibited at 5 mM and 10 mM nitrite. This dose-dependent response indicates that overexpression of ScdA renders *E. coli* more vulnerable to nitrite stress, presumably due to enhanced NO production. Data are presented as mean ± standard error (n>=6 independent experiments).

(**B**) *E. coli* cell viability assay using an empty pET-28a vector that lacks the *scdA* gene. All other experimental conditions were identical to those in (**A**). Under these conditions, *E. coli* growth remains largely unaffected by the increasing nitrite concentrations, confirming that the concentrations of nitrite used in this study are not inherently toxic to normal *E. coli* cells. Taken together, these findings support the conclusion that the growth inhibition observed in (**A**) and Figure 7 is specifically attributable to ScdA-enhanced NO production rather than general nitrite toxicity. Data are presented as mean ± standard error (n>=4 independent experiments).

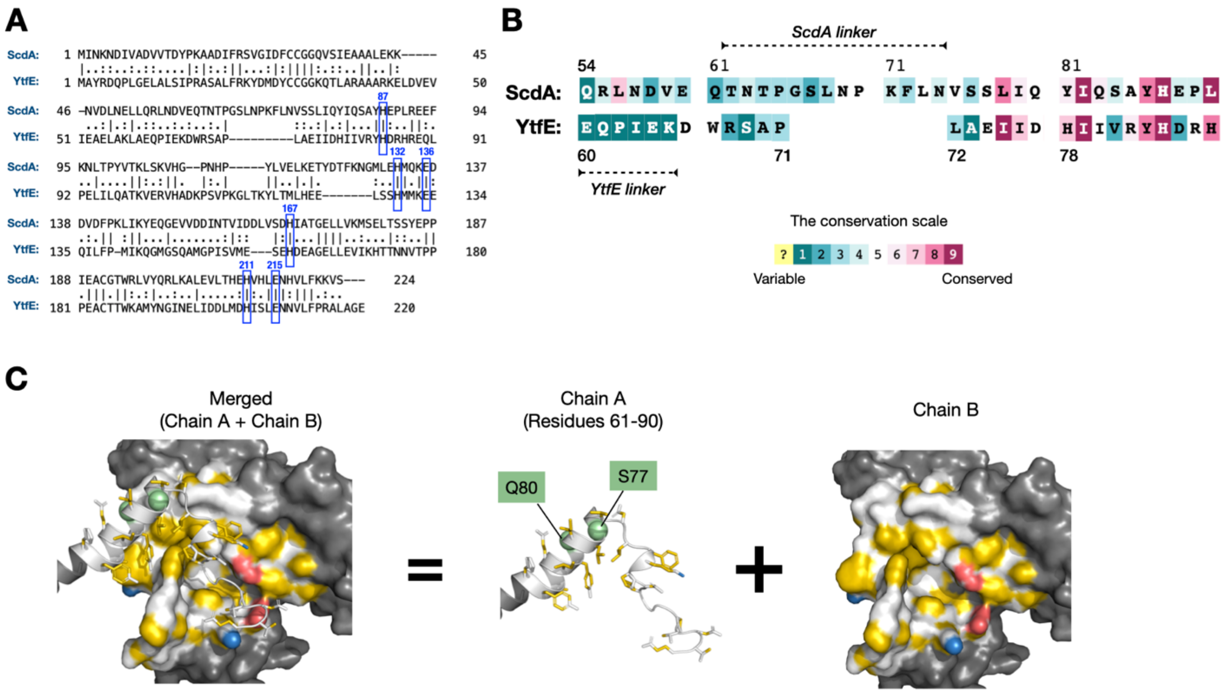

Figure S9. Sequence Alignment, ConSurf and YRB Analyses of the Dimer Interface.

(**A**) Sequence alignment of *S. aureus* ScdA and *E. coli* YtfE homologs. The alignment reveals a modest sequence identity of 27.5% (66/240 residues) and a sequence similarity of 47.5% (114/240 residues). Conserved residues are indicated by vertical bars (|), while chemically similar substitutions are marked by colons (:) and dots (.). Despite the moderate sequence conservation, both proteins share a two-domain architecture and conserve key residues (blue boxes) involved in the coordination of the diiron center, which is critical for their biochemical function.

(**B**) ConSurf comparison of ScdA and YtfE, highlighting the substantial variability in their linker regions. In ScdA, residues 62–74 form a longer linker than the 60–65 segment in YtfE. Both linkers display high sequence variability across RIC homologs, indicating that these regions are under less evolutionary constraint compared to the di-iron center.

(**C**) YRB color mapping of the linker interface in the ScdA dimer, where hydrophobic atoms appear yellow and charged residues appear blue (positive) or red (negative). Six non-polar residues (e.g., Pro, Gly, Leu, Phe) create a hydrophobic patch that interlocks with a corresponding pocket in the partner monomer, stabilizing dimer formation. Additionally, the orientation of S77 and Q80 favors inter-ScdA hydrogen bonding, showing how even a seemingly variable linker can support stable dimerization in ScdA.

Table S1 Tagless ScdA Data collection and refinement statistics (molecular replacement)

	**Tagless CF ScdA**
**Data collection**	
Wavelength (Å)	0.99987 Å
Space group	$P\ 2_12_12_1$
Cell dimensions	
a, *b*, *c* (Å)	69.54, 92.49, 110.89
α, β, γ (°)	90, 90, 90
Resolution (Å)	28.07-2.38 (2.44-2.38)*
R_{merge}	0.073 (0.765)*
$I / \sigma(I)$	13.8 (2.0)*
Completeness (%)	99.5 % (96.3%)*
Redundancy	6.5 (5.9)*
**Refinement**	
Resolution (Å)	28.07-2.38 (2.44-2.38)*
No. reflections	29,186
R_{work} / R_{free}	0.218/0.2522
No. atoms	
Protein	2,582
Ligand/ion	6 (2 Oxygen atoms, 4 Fe ions)
B-factors	
Protein	64.46
Ligand/ion	62.9
R.m.s. deviations	
Bond lengths (Å)	0.5
Bond angles (°)	0.58

*Values in parentheses are for highest-resolution shell.
